# A survey of Phrurolithidae spiders from Jinggang Mountain National Nature Reserve, Jiangxi Province, China

**DOI:** 10.3897/zookeys.947.51175

**Published:** 2020-07-08

**Authors:** Ke-Ke Liu, Hui-Pu Luo, Yuan-Hao Ying, Yu-Xin Xiao, Xiang Xu, Yong-Hong Xiao

**Affiliations:** 1 College of Life Science, Jinggangshan University, Ji’an 343009, Jiangxi, China Jinggangshan University Ji'an China; 2 College of Life Science, Hunan Normal University, Changsha 410081, Hunan, China Hunan Normal University Changsha China

**Keywords:** Taxonomy, new species, *Alboculus* gen. nov., *
Otacilia
*

## Abstract

Phrurolithidae spiders were collected from Jinggang Mountain National Nature Reserve, Jiangxi Province, China, during the past six years. The new genus *Alboculus* Liu, **gen. nov.**, with the type species *Phrurolithus zhejiangensis* Song & Kim, 1991, is described, and its previously unknown male is described for the first time. Furthermore, seven new species of *Otacilia* are described: *O. acutangula* Liu, **sp. nov.** (♂♀), *O. bijiashanica* Liu, **sp. nov.** (♂♀), *O. longtanica* Liu, **sp. nov.** (♀), *O. ovoidea* Liu, **sp. nov.** (♂♀), *O. shenshanica* Liu, **sp. nov.** (♂♀), *O. subovoidea* Liu, **sp. nov.** (♂♀), and *O. xiaoxiica* Liu, **sp. nov.** (♀). All species are illustrated with photographs and their distributions are mapped.

## Introduction

*Otacilia* was established by Thorell (1897), with the type species *O. armatissima* Thorell, 1897 from Myanmar (Burma). In the past ten years, the total number of species in this genus has increased greatly, approximately tripling, with many new species being discovered particularly from China (WSC 2020). Recently, after 27 *Phrurolithus* C.L. Koch, 1839 species were transferred to *Otacilia* (Zamani and Marusik 2020), the genus became the most diverse group of the 14 phrurolithid genera, currently including 99 of the 231 described phrurolithid species (WSC 2020). To date, there are 74 *Otacilia* species reported from China (ca. 75% of the total; WSC 2020). However, there are still many poorly known Phrurolithidae species from southern China with unusual morphological characteristics.

Even in 2020, there is no clear way of differentiating between the genera *Otacilia* and *Phrurolithus*, although some taxonomists have tried to do so (e.g., Wang et al. 2015; Fu et al. 2016; Jin et al. 2016; Liu et al. 2019). Detailed morphological characteristics of the genus *Phrurolithus* were not revealed until the study of Zamani and Marusik (2020), wherein many previously undocumented characters on the palps and epigynes were described for the first time. Many species described in *Phrurolithus* were incorrectly attributed to this genus, including the Chinese species, which were all transferred to *Otacilia* recently (Zamani and Marusik 2020). Only a few taxonomic works were published in recent years, but *Otacilia* has not been subjected to a comprehensive revision yet.

While studying spiders from Jinggang Mountain National Nature Reserve, Jiangxi Province, China, we found several phrurolithid spiders belonging to unknown species or undescribed sexes in the past six years. The male of *Otacilia zhejiangensis* (Song & Kim, 1991) was firstly recognised as the undescribed conspecific sex of this species. *Alboculus* Liu gen. nov. is proposed here based on the male and female of *O. zhejiangensis*. Furthermore, seven new *Otacilia* species are described in the present study.

## Materials and methods

Specimens were examined using a Zeiss Stereo Discovery V12 stereomicroscope with a Zoom Microscope System. Both male palps and female copulatory organs were detached and examined in 75% ethanol, using a Zeiss Axio Scope A1 compound microscope with a KUY NICE CCD. The epigynes were digested and cleared with pancreatin. Specimens including detached male palps and epigynes were stored in 80% ethanol after examination. All the specimens are deposited in Animal Specimen Museum, Life Science of College, Jinggangshan University (**ASM-JGSU**).

Somatic morphological measurements were taken with the ImageView CM2000 software and given in millimetres. The body length of all specimens excludes the chelicerae and spinnerets. Terminology of the male and female genitalia follows Jäger and Wunderlich (2012), Ramírez (2014), and Zamani and Marusik (2020). Promarginal and retromarginal teeth on the chelicerae are given as the first, second, third, etc., and measured from the base of the fang to the distal groove.

Leg measurements are given as total length (femur, patella, tibia, metatarsus, tarsus). Leg spines are documented by dividing each leg segment into two aspects, prolateral (p) and retrolateral (r), and indicating the ventral (v) spines as single (1) or paired (2), e.g., femur I pv1111; tibia I v2222.

The abbreviations used in the text are as follows:

**Eyes**

**ALE** anterior lateral eye;

**AME** anterior median eye;

**MOA** median ocular area;

**PLE** posterior lateral eye;

**PME** posterior median eye.

**Chelicerae**

**PES** promarginal escort seta;

**PRS** promarginal rake setae;

**RES** retromarginal escort seta;

**SS** slit sensillum;

**WS** whisker setae.

**Legs**

**LO** lyriform organ;

**MTS** metatarsal stopper;

**TO** tarsal organ.

**Male palp**

**DTA** dorsal tibial apophysis;

**dTA** distal tegular apophysis;

**E** embolus;

**FA** femoral apophysis;

**RTA** retrolateral tibial apophysis;

**rTA** retrolateral tegular apophysis;

**SD** sperm duct.

**Epigyne**

**B** bursa;

**CD** copulatory duct;

**CO** copulatory opening;

**CT** connecting tube;

**FD** fertilisation duct;

**GA** glandular appendage;

**MS** median septum;

**SP** spermathecae.

## Taxonomy

### Family Phrurolithidae Banks, 1892

**Comments.**Phrurolithidae spiders are mainly distributed in Asia, North America and Europe. Half of them are found from Asia. Four phrurolithid genera are Asian endemics, i.e., *Abdosetae* Fu, Zhang & MacDermott, 2010, *Bosselaerius* Zamani & Marusik, 2020, *Otacilia* Thorell, 1897 and *Plynnon* Deeleman-Reinhold, 2001. Only one genus, *Phrurolithus*, is widely distributed in Asia, America and Europe. Currently, more than 80 known species in the four former genera have been reported from China. The total number of known Phrurolithidae species from China will rapidly rise to 100 with the addition of seven new species described in the present paper and the future descriptions of additional new species from the country.

#### Alboculus

Taxon classificationAnimaliaAraneaePhrurolithidae

Liu gen. nov.

C8F9ADC3-6638-5EE7-ACBC-3287ADFC9C49

http://zoobank.org/3EF7496B-294B-4683-9887-C4367E06BA63

##### Diagnosis.

**Figure 1.**
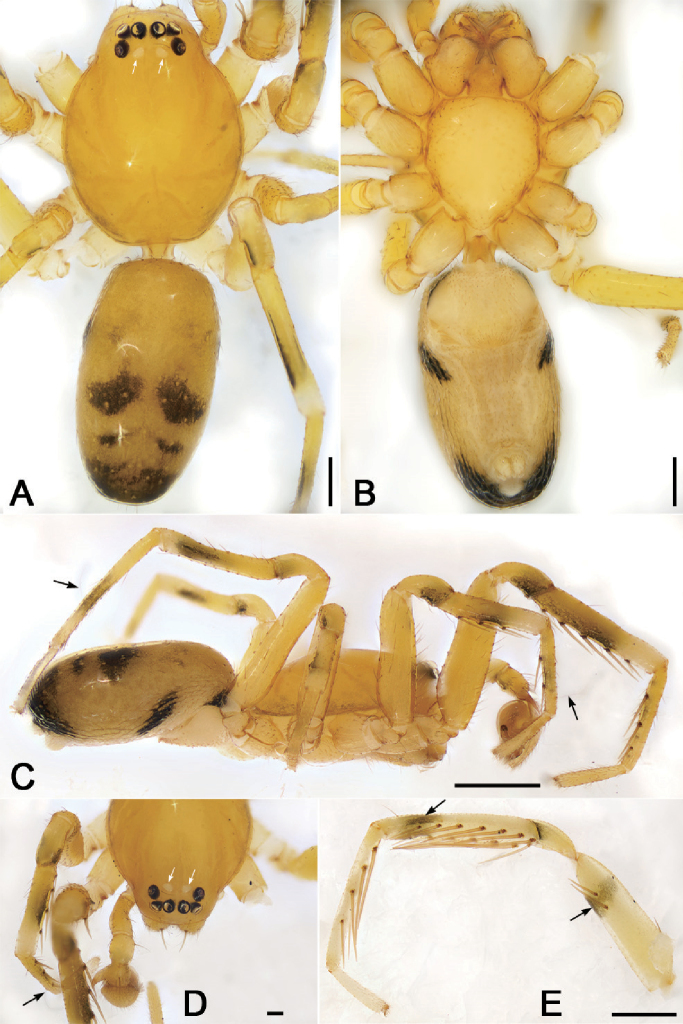
*Alboculus zhejiangensis* (Song & Kim, 1991) comb. nov., male **A** habitus, dorsal view, white arrows show the light-coloured, oval posterior median eyes **B** same, ventral view **C** same, lateral view, black arrows showing the long trichobothria on metatarsi II and IV **D** carapace, dorsal view, white arrows show the light-coloured, oval posterior median eyes, black arrow shows the long trichobothrium on metatarsus II **E** right leg I, prolateral view, black arrows showing the dark annulations. Scale bars: 0.2 mm (**A, B**), 0.5 mm (**C, E**), 0.1 mm (**D**).

**Figure 2.**
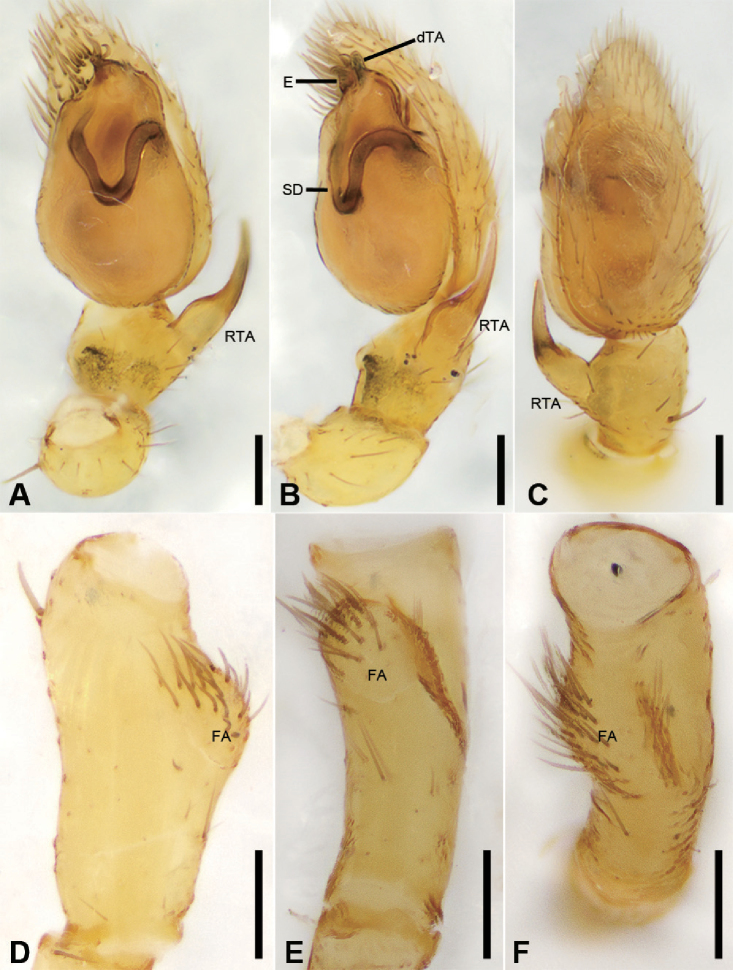
*Alboculus zhejiangensis* (Song & Kim, 1991) comb. nov., male palp **A** palp, prolateral view **B** same, ventral view **C** same, retrolateral view **D** femur, prolateral view **E** same, ventral view **F** same, retrolateral view. Scale bars: 0.2 mm (**A, B**), 0.1 mm (**C–F**). Abbreviations: dTA – distal tegular apophysis, E – embolus, FA – femoral apophysis, RTA – retrolateral tibial apophysis, SD – sperm duct.

**Figure 3.**
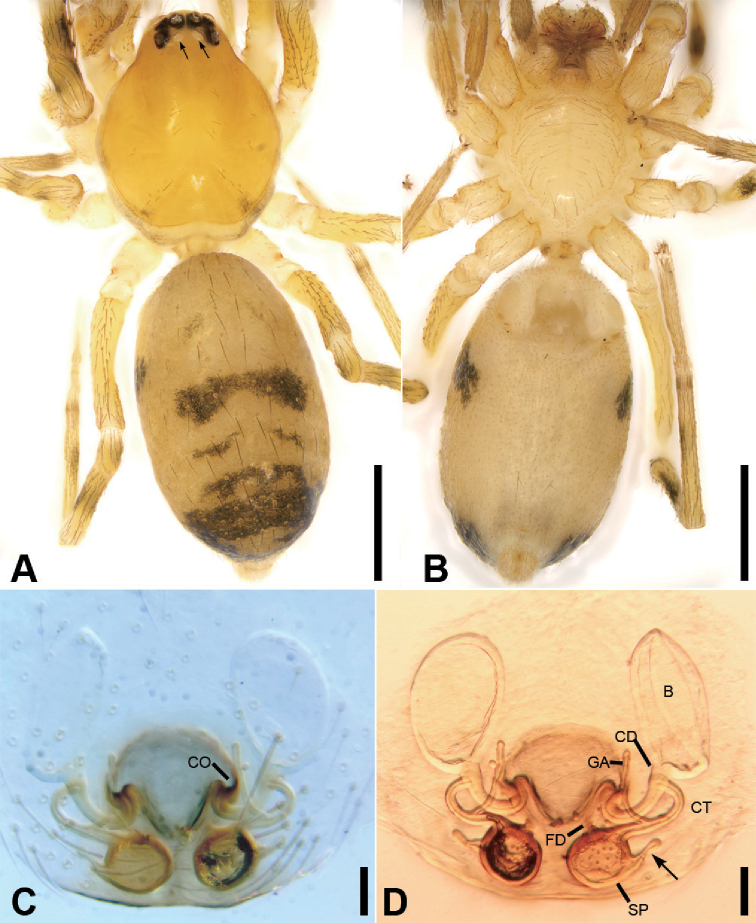
*Alboculus zhejiangensis* (Song & Kim, 1991) comb. nov., female **A** habitus, dorsal view, black arrows show the light-coloured, oval posterior median eyes **B** same, ventral view **C** epigyne, ventral view **D** same, dorsal view, black arrow shows the detail of spermathecal tail. Scale bars: 0.5 mm (**A, B**), 0.1 mm (**C, D**). Abbreviations: B – bursa, CD – copulatory duct, CO – copulatory opening, CT – connecting tube, FD – fertilisation ducts, GA – glandular appendage, SP – spermathecae.

The new genus differs from other Phrurolithidae by the oval PME without a layer of black pigment around the eye cup (Figs 1A, D, 3A) (vs. with layer of black pigment around eye cup), posterior eye row slightly procurved (Figs 1A, D, 3A) (vs. straight to recurved), lacking distinct longitudinal and radial stripes on the dorsal carapace (Figs 1A, D, 3A) (vs. black longitudinal or radial stripes present), and lacking a chevron-shaped marking on the abdominal dorsum (Figs 1A, 3A) (vs. with at least two chevron-shaped markings). Males of this genus can be easily distinguished by the lack of a dorsal tibial apophysis on the palp (Figs 2A‒C, 6A, B, D) (vs. palpal tibia with dorsal tibial apophysis) and the well-developed terminal apophysis of the bulb (Figs 2A‒C, 6B‒D) (vs. absent). The female of this genus has the glandular appendages slender (Fig. 3C, D) (vs. relatively short and thick) and the spermathecal tail of epigyne distinct (Fig. 3D, E) (vs. without a spermathecal tail).

##### Type species.

*Otacilia zhejiangensis* (Song & Kim, 1991).

##### Etymology.

The genus name is formed from two Latin words *albus* and *oculus*, alluding to the light-coloured posterior median eyes; the gender is masculine.

##### Remarks.

The type species *O. zhejiangensis* was first described by Song and Kim (1991) as a new species of *Phrurolithus* based on a single female specimen from Tianmu Mountain, Zhejiang province, China. Recently, it was transferred to *Otacilia* by Zamani and Marusik (2020). It is interesting to compare the three specimens of this species, clearly recognised by differences in morphological characters with the type species of *Otacilia* and *Phrurolithus*. Males of this genus differ from *Phrurolithus festivus* (C.L. Koch, 1835) by lacking a layer of black pigment around the PME (Figs 1A, B, D, 3A) (vs. PME with black pigment), and having a single tibial apophysis (Figs 2A‒C, 6A, B, D) (vs. present two tibial apophysis). Although the male of *Otacilia armatissima* is unknown, male *Alboculus* species differ from *Otacilia* males (e.g., Figs 7A, 9A, 13A, 15A, 18A) by the procurved posterior eye row (vs. recurved), and by the dorsal scutum covering the entire dorsal surface of the abdomen (Fig. 1A, C) as opposed to a narrow scutum only extending to approximately half the abdomen length in *Otacilia* (e.g., Figs 7A, 9A). The females clearly differ from these two type species (*O. armatissima* and *P. festivus*) by the slender glandular appendages (Fig. 3C, D) (vs. relatively short and thick [Figs 8D, 10D, 12D, 14D, 16D, 19D, 21D]) and the spermathecal tail of epigyne (Fig. 3C, D) (vs. without the spermathecal tail [Figs 8D, 10D, 12D, 14D, 16D, 19D, 21D]).

##### Description.

Small, body length 1.8‒2.8 mm. ***Eyes***: AME rounded, PME oval, light-coloured, without black pigment, anterior eye row straight, posterior eye row procurved. Each chelicera with three promarginal and two retromarginal teeth. Femur I with wo spines, tibia I with five pairs of ventral spines, metatarsus I with three pairs of ventral spines. Abdomen without dorsal scutum in females, covering entire dorsum in males.

***Male palp***: femur with large ventral extension; tibia with long, sharply-pointed retroventral tibial apophysis, without dorsal apophysis; bulb without median apophysis or conductor; sperm duct long, reaching middle part of the tegulum, narrowed near base of embolus; base of embolus slightly narrowed, embolus very small, hook-shaped, directed antero-prolaterally, embolus accompanied by thick, short distal terminal apophysis (TA) (larger than embolus). Epigyne with clear copulatory atrium medially; glandular appendages slender, located on anterior of connecting tubes; spermathecae rounded, with clavate-like tail.

##### Distribution.

**Map 1.**
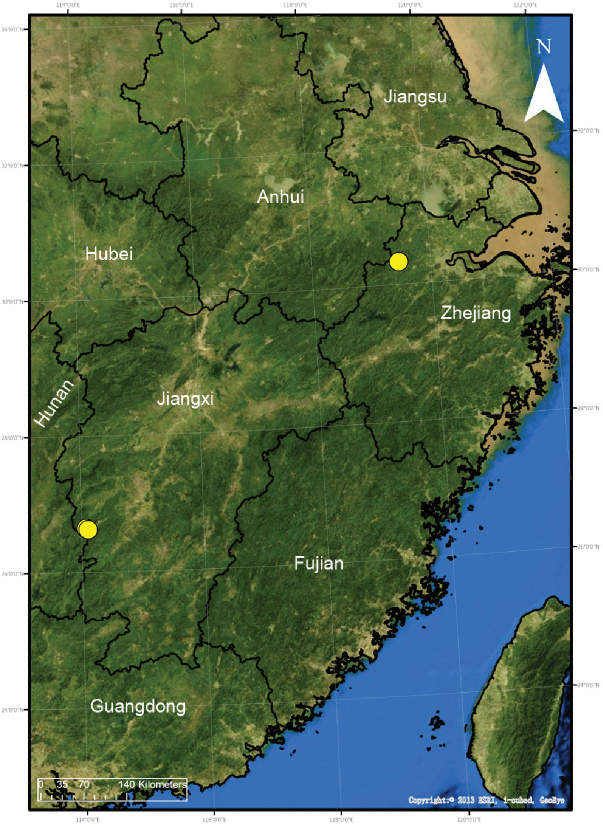
Distribution of *Alboculus zhejiangensis* (Song & Kim, 1991), comb. nov., in China.

China (Map 1) (Zhejiang and Jiangxi Provinces)

**Figure 4.**
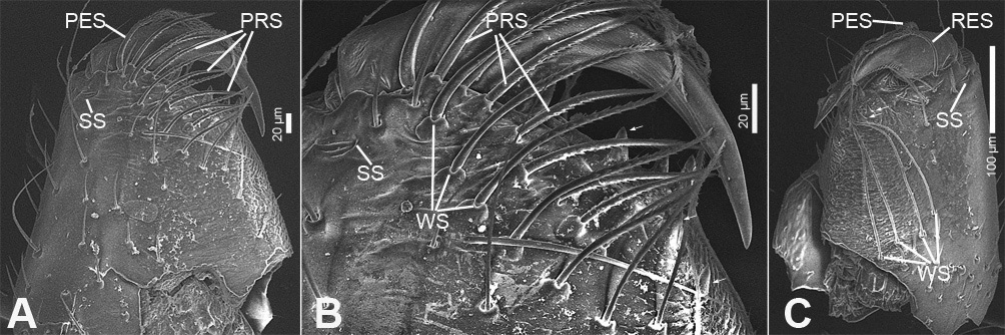
SEM micrographs of *Alboculus zhejiangensis* (Song & Kim, 1991) comb. nov., male chelicera **A** frontal view **B** detail of promargin, frontal view **C** posterior view, slightly retrolateral. Abbreviations: PES – promarginal escort seta, PRS – promarginal rake setae, RES – retromarginal escort seta, SS – slit sensillum, WS – whisker setae.

#### Alboculus zhejiangensis

Taxon classificationAnimaliaAraneaePhrurolithidae

(Song & Kim, 1991) comb. nov.

9C93703B-8727-5671-86C5-95F64BB7F6EC

**Figure 5.**
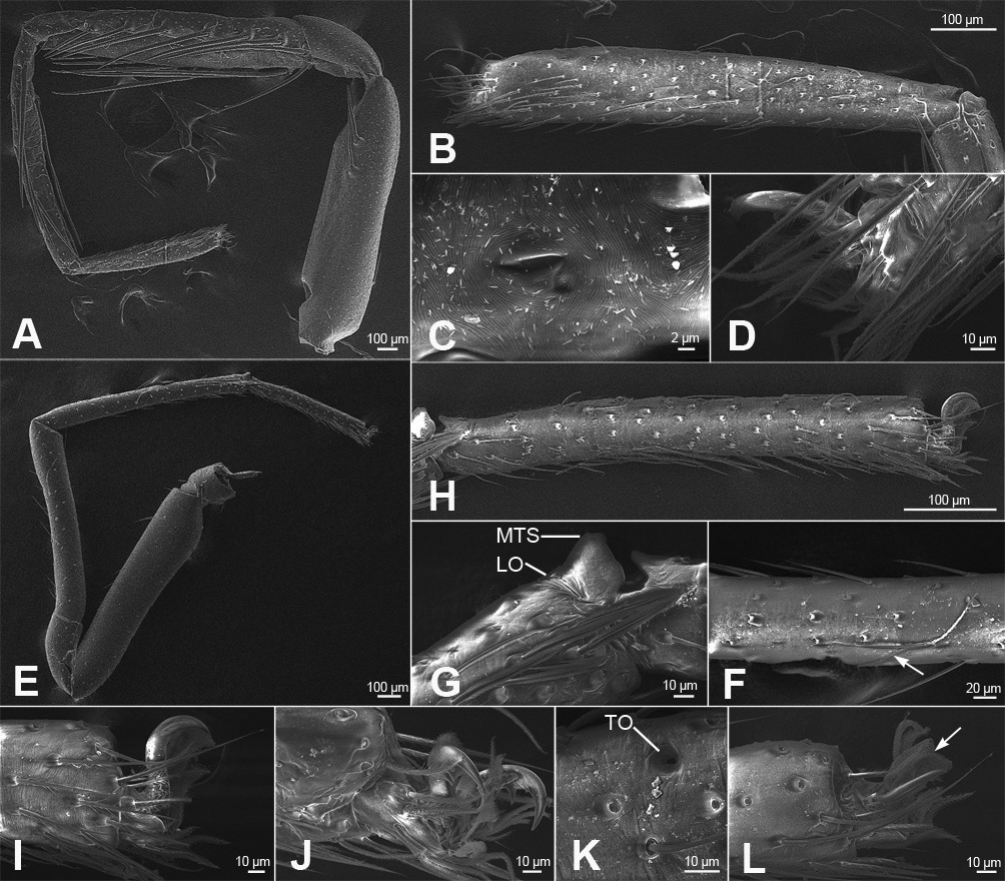
SEM micrographs of *Alboculus zhejiangensis* (Song & Kim, 1991) comb. nov., male legs **A** right leg I, prolateral view **B** same, tarsus, prolateral view **C** same, tarsal slit sensillum, prolateral view **D** same, tarsal claw and claw tuft setae, prolateral view **E** left leg IV, prolateral view **F** same, metatarsus, white arrow shows the long trichobothrium, prolateral view **G** same, metatarsus-tarsus joint, prolateral view **H** same, tarsus, prolateral view **I** same, tarsal claw and claw tuft setae, prolateral view **J** right tarsal claw I and claw tuft setae, retrolateral view **K** left tarsus IV, detail of tarsal organ, dorsal view **J** left tarsal claw IV and claw tuft setae, dorsal view. Abbreviations: LO – lyriform organ, MTS – metatarsal stopper, TO – tarsal organ.

**Figure 6.**
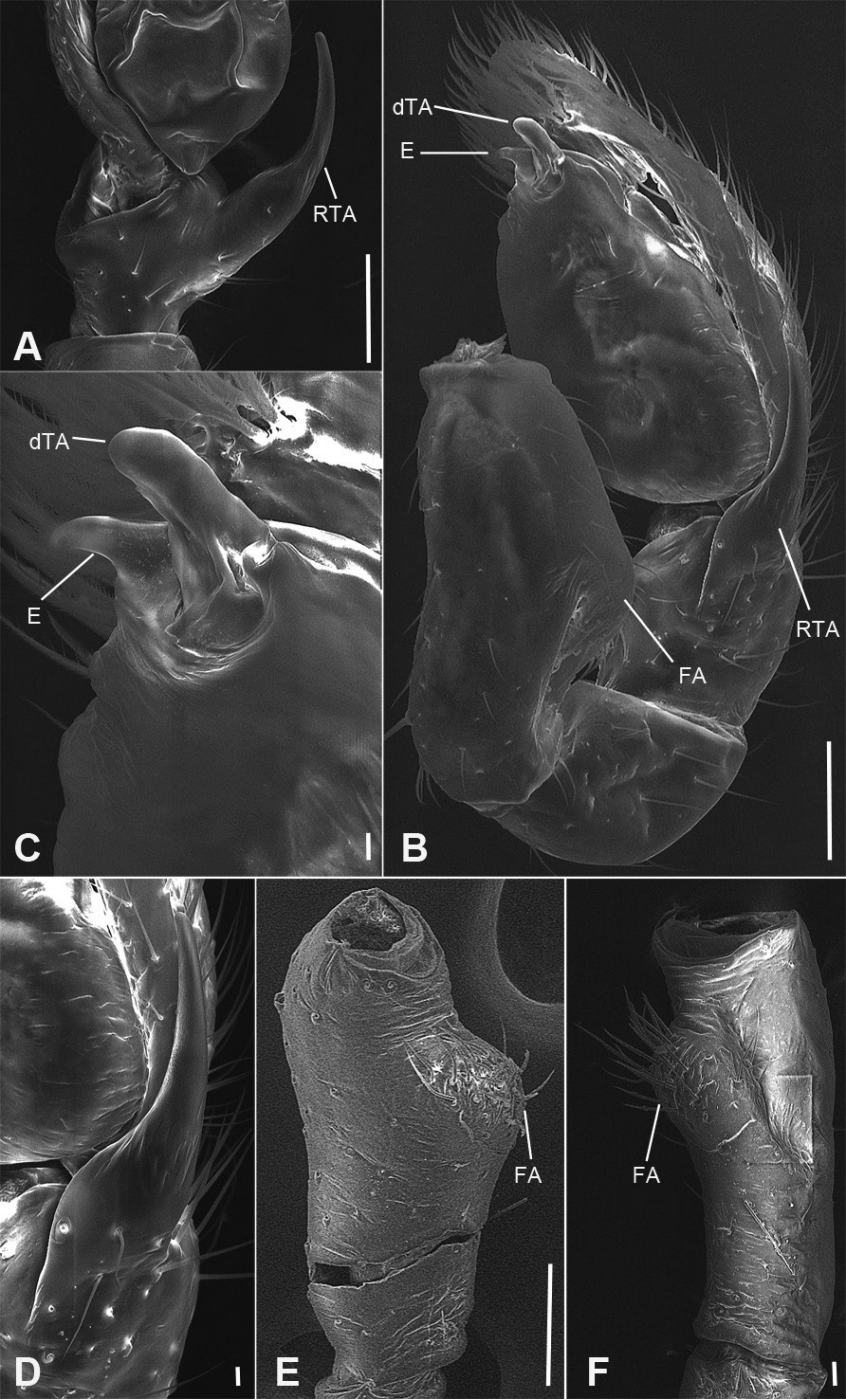
SEM micrographs of *Alboculus zhejiangensis* (Song & Kim, 1991) comb. nov., male palp **A** ventral view, showing detail of retrolateral tibial apophysis **B** same, ventro-retrolateral view **C** same, detail showing embolus and distal tegular apophysis **D** same, detail showing retrolateral tibial apophysis **E** palpal femur, prolateral view **F** same, retrolateral view. Scale bars: 0.1 mm (**A, B, E**), 10 µm (**C, D**), 20 µm (**F**). Abbreviations: dTA – distal tegular apophysis, E – embolus, FA – femoral apophysis, RTA – retrolateral tibial apophysis.

Figures 1 , 2 , 3 , 4 , 5 , 6

Phrurolithus zhejiangensis Song & Kim, 1991: 23, figs 16–18 (♀); Song et al. 1999: 412, fig. 240E–F (♀).
Otacilia zhejiangensis Zamani & Marusik, 2020: 312.

##### Material examined.

China: Jiangxi Province, Ji’an City, Jinggangshan County Level City. 2♂, Dalong Town, Yuantou Village, 26°37'40.8"N, 114°6'21.6"E, 906 m, 5 April 2014, leg. Ke-Ke Liu et al.; 1♀, Longshi Town, Maoping, Shenshan Village, Shenshan, 26°38'49.2"N, 114°4'26.4"E, 798 m, 8 August 2015, leg. Ke-ke Liu et al.

##### Notes.

These two collection localities of males and a female of this species are very close and located on both sides of Shenshan Mt. They are assigned in different two adjacent towns in Jinggang Mountain National Nature Reserve, Jiangxi Province, China. Meanwhile, one sub-adult male was also collected on 8 August 2015, which has the same habitus as the males collected on April 5 2014. These males are therefore recognised as corresponding to the conspecific female.

##### Diagnosis.

This species is easily distinguished from other Phrurolithidae spiders by the following combination of morphological characteristics: (1) lacking a layer of black pigment around the PME (Figs 1A, D, 3A) (vs. PME with black pigment); (2) lacking distinct longitudinal and radial stripes on the dorsal carapace (Figs 1A, D, 3A) (vs. black longitudinal or radial stripes present); (3) lacking chevron-shaped marking on abdominal dorsum (Figs 1A, 3A) (vs. with at least two chevron-shaped markings); (4) male palpal tibia with a single retrolateral apophysis (Figs 2A–C, 6A, B, D) (vs. two tibial apophyses present); (5) female epigyne (Fig. 3C, D) with the glandular appendages slender (vs. relatively short and thick), and the spermathecal tail club-shaped (vs. without a spermathecal tail).

##### Description.

**Male.** Habitus as in Fig. 1A−C. Total length 2.50, carapace 1.18 long, 0.90 wide. ***Eye*** sizes and interdistances: AME 0.06, ALE 0.07, PME 0.06, PLE 0.06; ALE−AME 0.02, AME–AME 0.04, PLE−PME 0.06, PME–PME 0.06, ALE−ALE 0.21, PLE−PLE 0.28, ALE−PLE 0.05, AME−PME 0.06, ALE−PME 0.11. MOA 0.17 long, front width 0.17, back width 0.19. Cervical groove distinct. Radial furrow and fovea indistinct. ***Chelicerae*** (Figs 2A, B, 4): with two frontal spines long and short, three promarginal (proximal largest, distal smallest) and two retromarginal teeth (distal larger); promargin with one escort seta, a row of rake setae, a row of whisker setae; retromargin with one escort seta; the other row of whisker setae present near the cheliceral base in retrolateral view; near base of fang with a prolateral and a retrolateral slit sensillum. Sternum with strongly rebordered margins (Fig. 1B). Leg measurements: I 3.21 (0.94, 0.39, 0.87, 0.62, 0.39); II 2.67 (0.81, 0.36, 0.66, 0.49, 0.35); III 2.48 (0.67, 0.31, 0.55, 0.57, 0.38); IV 3.59 (0.96, 0.37, 0.82, 0.92, 0.52). ***Leg*** setae: metatarsi I, II, and IV with a long trichobothrium, as long as tarsus; tarsi I−IV with 2−4 trichobothria each; tarsal claws with 5−12 pseudotenent setae each, superior tarsal claw with two teeth. Tarsal organ teardrop shaped (Fig. 5K). Tarsal slit sensillum present. Leg spination: femur I pv11; tibiae I v22222, II v222; metatarsi I pv1111, rv111, II pv111, rv11. Abdomen elongate elliptical in dorsal view (Fig. 1A−C), scutum covering entire dorsum, 1.24 long, 0.69 wide.

***Colouration*** (Fig. 1A−C). Carapace yellow, with indistinct radial stripes from median to marginal. Chelicerae, endites, labium, and sternum yellow. Legs yellow, with dark strips on patellae, tibiae and metatarsi I−IV (Figs 1, 5). Abdomen yellow, with pair of large oval dark spots medially, pair of blade-shaped dark spots on sub-medial part, and semi-circular dark spot posteriorly.

***Palp*** (Figs 2, 6). Femoral apophysis well-developed, width slightly less than half of length, with abundant short setae. Patella unmodified. Tibia with a large retrolateral apophysis, longer than tibia, with sharply pointed and broad base. Cymbium approximately two times longer than wide. Bulb oval, with long V-shaped sperm duct, apophyses absent. Embolus hook-shaped, small, with large base, accompanied by a small tegular apophysis of embolic base, terminal apophysis slightly longer than embolus and surrounded by the embolic base.

**Female.** Habitus as in Fig. 3A, B. Total length 2.40, carapace (Fig. 3A) 1.01 long, 0.79 wide. ***Eye*** sizes and interdistances: AME 0.06, ALE 0.07, PME 0.05, PLE 0.05; ALE−AME 0.02, AME–AME 0.04, PLE−PME 0.03, PME–PME 0.04, ALE−ALE 0.17, PLE−PLE 0.20, ALE−PLE 0.06, AME−PME 0.06, ALE–PME 0.20. MOA 0.15 long, front width 0.14, back width 0.14. ***Abdomen*** (Fig. 3A), 1.08 long, 1.19 wide. ***Leg*** measurements: I 2.73 (0.76, 0.31, 0.71, 0.59, 0.36); II 2.15 (0.65, 0.27, 0.50, 0.49, 0.33); III 1.99 (0.58, 0.25, 0.37, 0.48, 0.31); IV 2.78 (0.79, 0.30, 0.60, 0.68, 0.41). Dorsal scutum absent on abdomen.

***Epigyne*** (Fig. 3C, D). Anterior fovea separated by weakly sclerotised V-shaped margin, bilaterally with concaved copulatory openings. Copulatory ducts and gland appendages distinctly visible through integument in intact epigyne. Copulatory ducts slender, curved forward, connecting with the oval bursae. Connecting tubes slender, ear-shaped, located at the distal of copulatory ducts, curved backwards to spermathecae, posteriorly with slender glandular appendages. Glandular appendages as long as connecting tubes, extending forwards. Spermathecae globular, separated less than their diameter. Fertilisation duct short, located anteriorly on spermathecae. Spermathecal tails shorter than spermathecal diameter, club-shaped, ectally located.

##### Distribution.

Known from Zhejiang and Jiangxi (Map 1).

#### Genus Otacilia

Taxon classificationAnimaliaAraneaePhrurolithidae

Thorell, 1897

DB734E67-872B-5677-8234-F23D563480E1

##### Notes.

Currently, there are 99 species included in this genus, with 74 recorded from China. In the last five years, the total number of species from the country has increased considerably, due to the considerable attention paid to them by many arachnologists. They are widely distributed in southern China, such as Hainan (six species), Taiwan (two species), Zhejiang (four species), Yunnan (ten species), Guangxi (two species), Guizhou (five species), Sichuan (eight species), Chongqing (nine species), Hunan (19 species), Hubei (four species) and Jiangxi (seven species) provinces. Jin et al. (2016) divided *Otacilia* into five species groups, i.e., the *armatissima*-group, *ambon*-group, *longituba*-group, *pseudostella*-group, and a fifth unnamed group containing the remaining species (i.e., species known from a single sex, or with poor original descriptions and figures or peculiar structures). These seven new species most likely belong to the *armatissima*-group. Only one new species, *O. bijiashanica* Liu, sp. nov., has two tibial apophyses, while the others only have one.

#### Otacilia acutangula

Taxon classificationAnimaliaAraneaePhrurolithidae

Liu sp. nov.

979F4C84-1F50-553A-97E8-D6E44F08B5CA

http://zoobank.org/B8364C5E-8AE3-4E18-BA7B-EF9D2D11454D

Figures 7 , 8

##### Type material.

***Holotype***: ♂, China: Jiangxi Province, Ji’an City, Jinggangshan County Level City, Ciping Town, Dajing Village, Jingzhushan Scenic Spot, 26°31'33.37"N, 114°06'30.34"E, 786 m, 1 October 2018, leg. Ke-Ke Liu et al. ***Paratypes***: 2♀, with same data as holotype; 1♂, 1♀, same locality, Lingxiufeng Scenic Spot, 26°34'16.72"N, 114°07'00.56"E, 971 m, 1 October 2018, leg. Ke-Ke Liu et al.; 1♂, same locality, Xiaojing Village, Longtan Scenic Spot, 26°35'33.08"N, 114°08'18.50"E, 909 m, 1 October 2018, leg. Ke-Ke Liu et al.; 1♂, same locality, Bijiashan Scenic Spot, Hongjun Road, 26°36'25.88"N, 114°11'43.07"E, 549 m, 3 October 2018, leg. Ke-Ke Liu et al.

##### Etymology.

The specific name is derived from the Latin adjective *acutangulus*, referring to the bent retrolateral tibial apophysis that forms an angle of ca. 45° with its transverse base; adjective.

**Figure 7.**
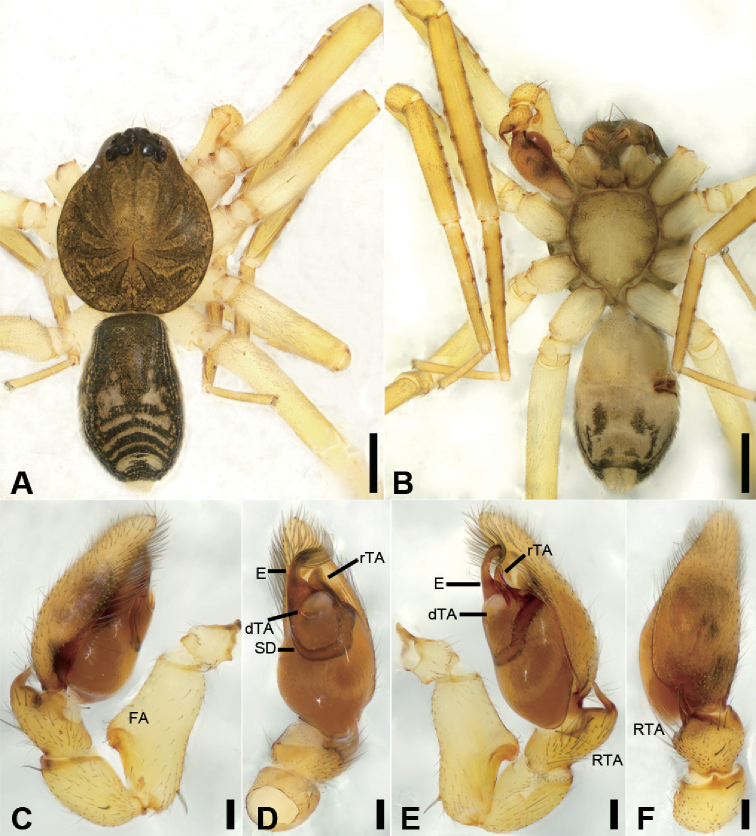
*Otacilia acutangula* sp. nov., male holotype **A** habitus, dorsal view **B** same, ventral view **C** palp, prolateral view **D** same, ventral view **E** same, retrolateral view **F** same, dorsal view. Scale bars: 0.5 mm (**A, B**), 0.1 mm (**C–F**). Abbreviations: dTA – distal tegular apophysis, E – embolus, FA – femoral apophysis, RTA – retrolateral tibial apophysis, rTA –retrolateral tegular apophysis, SD – sperm duct.

##### Differential diagnosis.

**Figure 8.**
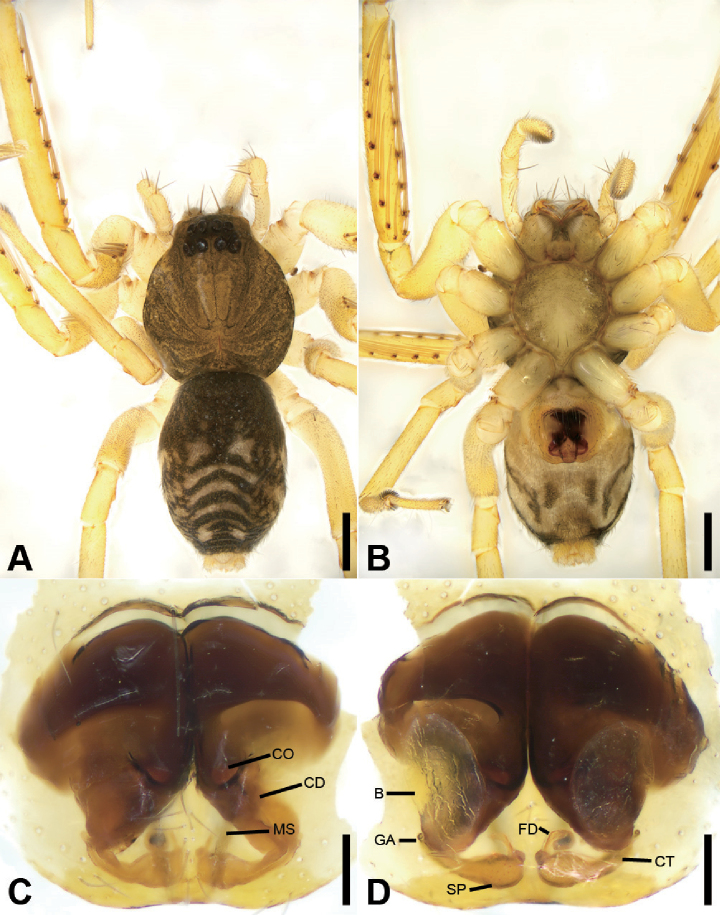
*Otacilia acutangula* sp. nov., female paratype **A** habitus, dorsal view **B** same, ventral view **C** epigyne, ventral view **D** epigyne, dorsal view. Scale bars: 0.5 mm (**A, B**), 0.1 mm (**C, D**). Abbreviations: B – bursa, CD – copulatory duct, CO – copulatory opening, CT – connecting tube, FD – fertilisation ducts, GA – glandular appendage, MS – median septum, SP – spermathecae.

The new species differs from *O. daweishan* Liu, Xu, Xiao, Yin & Peng, 2019 by an oval distal tegular apophysis (Fig. 7C−F) (vs. teardrop shaped), the bent RTA forming an angle of ca. 45° (Fig. 7D, E) (vs. ca. 60°), and the strongly sclerotised ridges in the epigyne (Fig. 8C) (vs. weakly sclerotised).

##### Description.

**Male** (Holotype). Habitus as in Fig. 7A, B. Total length 3.10, carapace 1.45 long, 1.31 wide. ***Eye*** sizes and interdistances: AME 0.08, ALE 0.10, PME 0.07, PLE 0.11; ALE−AME 0.02, AME–AME 0.06, PLE−PME 0.07, PME–PME 0.12, ALE−ALE 0.25, PLE−PLE 0.39, ALE−PLE 0.10, AME−PME 0.10, ALE−PME 0.10. MOA 0.25 long, front width 0.20, back width 0.27. ***Chelicerae*** (Fig. 7A, B) with three promarginal (middle largest, distal smallest) and five retromarginal teeth (distal largest, proximal smallest). ***Sternum*** (Fig. 7B), posteriorly pointed. ***Abdomen*** (Fig. 7A) 1.43 long, 0.91 wide. ***Leg*** measurements: I 6.64 (1.73, 0.57, 1.98, 1.48, 0.88); II 5.42 (1.38, 0.50, 1.52, 1.26, 0.76); III 4.57 (1.16, 0.50, 1.07, 1.15, 0.69); IV 7.15 (1.96, 0.55, 1.73, 1.95, 0.96). Leg spination: femora I–IV with one dorsal spine each; femora I pv1111, II pv11; tibiae I v22222222, II v2222222; metatarsi I v2222, II pv1222.

***Colouration*** (Fig. 7A, B). Carapace yellow-brown. Chelicerae yellow-brown. Endites yellow. Labium and sternum yellow-brown. Legs yellow. Abdomen yellowish brown, with pair of small oval large triangular yellowish spots medially, large irregular yellowish spots medially also on the posterior dorsal scutum, three light chevron-shaped stripes on sub-medial part, and yellowish arc-shaped stripe posteriorly; weak dorsal scutum in anterior half, extending slightly past the midpoint.

***Palp*** (Fig. 7C−F). Femoral apophysis well-developed, width longer than half of length. Patella unmodified. Tibia with short retrolateral apophysis, less than tibial length, tapering-pointed, bending inwards to base of cymbium, forming an acute angle of ca. 45° with its transverse base in retrolateral view. Cymbium more than two times longer than wide. Bulb oval, with long U-shaped sperm duct, apophyses absent. Embolus hook-shaped, thick, with broad triangular base, apart from distal and retrolateral tegular apophyses. Retrolateral tegular apophysis straight, thickened, finger-shaped, submedial part covered by oval distal tegular apophysis.

**Female.** Habitus as in Fig. 8A, B. Lighter than male. Total length 2.87, carapace 1.42 long, 1.20 wide. ***Eye*** diameters: AME 0.09, ALE 0.09, PME 0.07, PLE 0.09; ALE−AME 0.01, AME–AME 0.04, PLE−PME 0.06, PME–PME 0.11, ALE−ALE 0.23, PLE−PLE 0.35, ALE−PLE 0.08, AME−PME 0.09, ALE−PME 0.10. MOA 0.23 long, front width 0.21, back width 0.24. ***Abdomen*** (Fig. 8A) 1.37 long, 0.86 wide. ***Leg*** measurements: I 6.41 (1.62, 0.49, 2.00, 1.58, 0.72); II 5.27 (1.35, 0.53, 1.45, 1.22, 0.72); III 4.36 (1.15, 0.42, 1.00, 1.15, 0.64); IV 7.07 (1.88, 0.55, 1.75, 1.95, 0.94). Leg spination: femora I–IV with one dorsal spine each; tibiae I v2222222, II v222222.

***Epigyne*** (Fig. 8C, D). Epigynal plate mushroom-like, posterior with a triangular median septum, copulatory ducts, glandular appendages, connecting tubes and spermathecae distinctly visible through integument in intact epigyne. Anterior fovea separated by strongly sclerotised M-shaped margin, medially with concaved, large copulatory openings. Copulatory ducts broad, declivitous, posteriorly with pair of kidney-shaped transparent bursae medially. Glandular appendages short, anterior part covered by bursae, located on anterior of connecting tubes. Connecting tubes short, located between glandular appendages and spermathecae. Spermathecae slightly swollen, slightly separated. Fertilisation duct short, located apically on spermathecae.

##### Distribution.

**Map 2.**
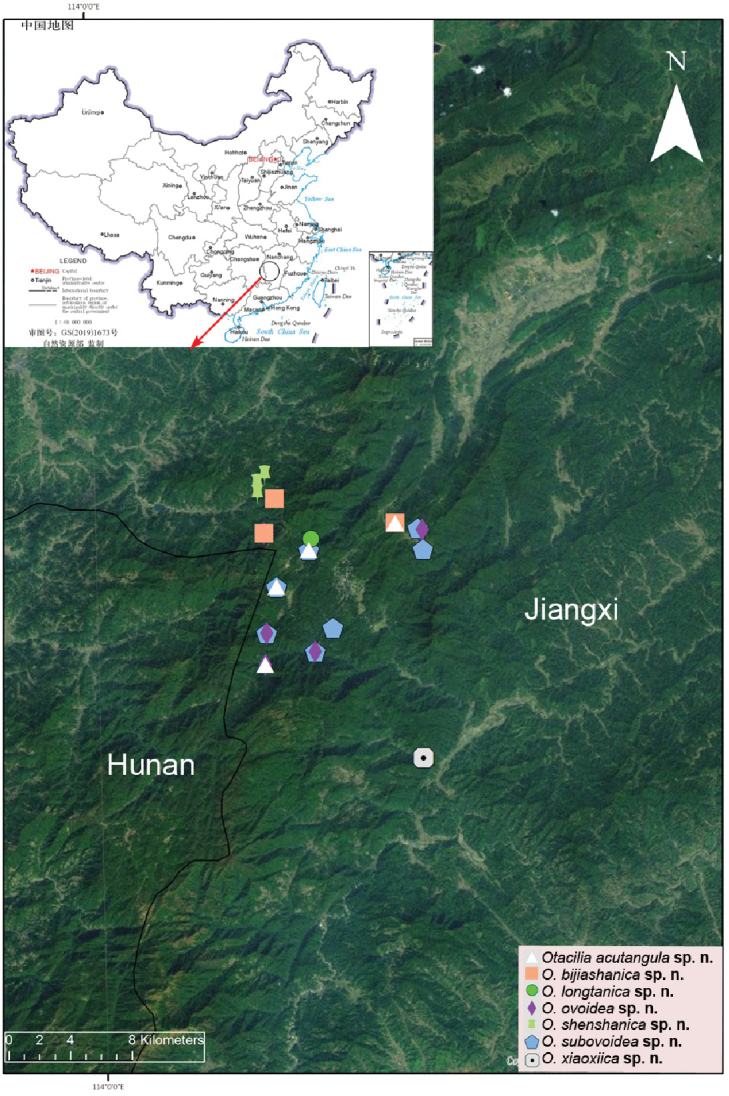
Map of China, enlargement showing records of *Otacilia acutangula* sp. nov., *O. bijiashanica* sp. nov., *O. longtanica* sp. nov., *O. ovoidea* sp. nov., *O. shenshanica* sp. nov., *Otacilia subovoidea* sp. nov. and *O. xiaoxiica* sp. nov. in Jinggang Mountain National Nature Reserve, Jiangxi.

Known only from the type locality in Jiangxi Province, China (Map 2).

#### Otacilia bijiashanica

Taxon classificationAnimaliaAraneaePhrurolithidae

Liu sp. nov.

CA70E2C5-B7F1-5A03-AACC-5B826D5806F7

http://zoobank.org/467DE20C-2700-49F0-A52D-B98B136164AE

Figures 9 , 10 , 11

##### Type material.

***Holotype***: ♂, China: Jiangxi Province, Ji’an City, Jinggangshan County Level City, Ciping Town, Bijiashan Scenic Spot, Hongjun Road, 26°36'25.88"N, 114°11'43.07"E, 549 m, 3 October 2018, leg. Ke-Ke Liu et al. ***Paratypes***: 3♂, 1♀, same locality as holotype, Luofu Town, Xiangzhou Village, Fengshuping Group, 26°36'10.31"N, 114°06'34.69"E, 364 m, 5 October 2018, leg. Ke-Ke Liu and Hui-Pu Luo; 1♂, Ciping Town, Huangyangjie Scenic Spot, 26°37'22.8"N, 114°7'1.2"E, 1055 m, 5 April 2014, leg. Ke-Ke Liu et al.

##### Etymology.

The specific name refers to the type locality, Bijiashan; adjective.

##### Differential diagnosis.

**Figure 9.**
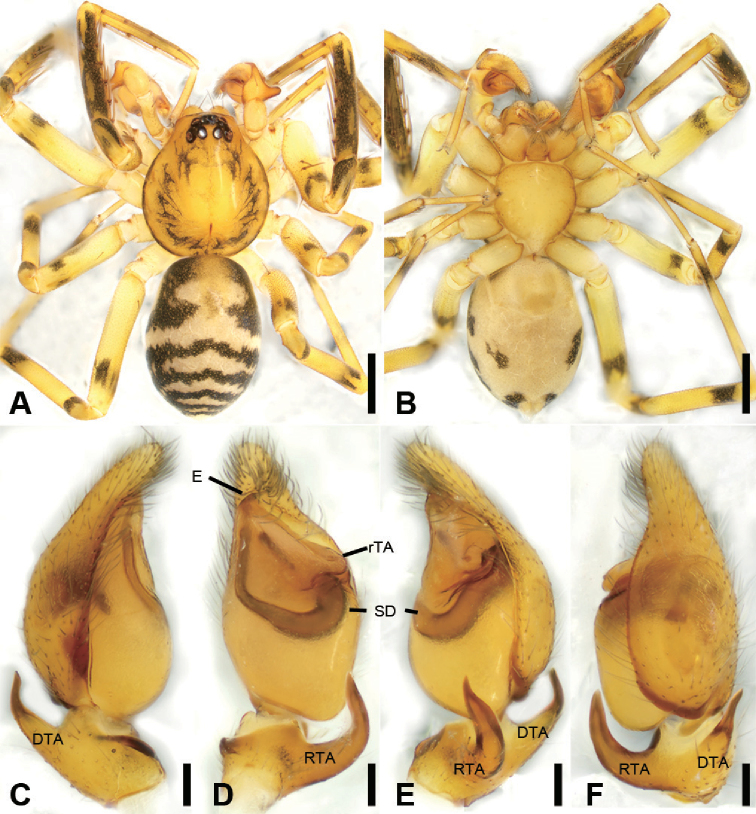
*Otacilia bijiashanica* sp. nov., male holotype **A** habitus, dorsal view **B** same, ventral view **C** palp, prolateral view **D** same, ventral view **E** same, retrolateral view **F** same, dorsal view, slightly retrolateral. Scale bars: 0.5 mm (**A, B**), 0.1 mm (**C–F**). Abbreviations: DTA – dorsal tibial apophysis, E – embolus, rTA – retrolateral tegular apophysis, RTA – retrolateral tibial apophysis, SD – sperm duct.

**Figure 10.**
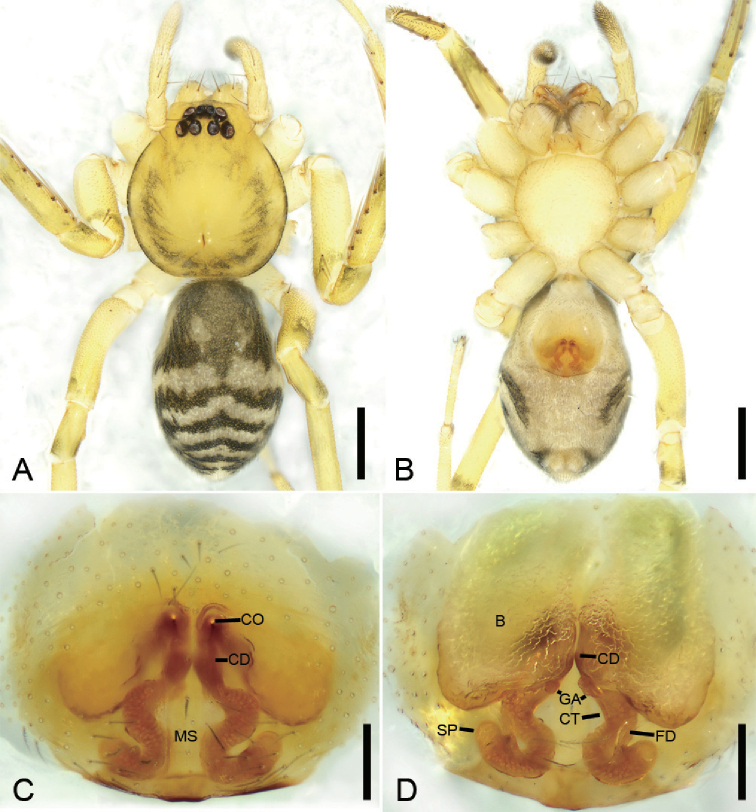
*Otacilia bijiashanica* sp. nov., female paratype **A** habitus, dorsal view **B** same, ventral view **C** epigyne, ventral view **D** epigyne, dorsal view. Scale bars: 0.5 mm (**A, B**), 0.1 mm (**C, D**). Abbreviations: B – bursa, CD – copulatory duct, CO – copulatory opening, CT – connecting tube, FD – fertilisation ducts, GA – glandular appendage, MS – median septum, SP – spermathecae.

**Figure 11.**
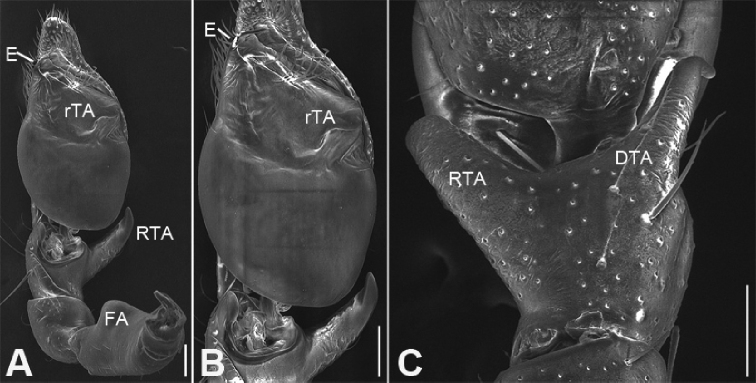
SEM micrographs of *Otacilia bijiashanica* sp. nov., palp of male paratype **A** ventral view **B** same, detail of bulb **C** dorsal view, detail of tibia apophysis. Scale bars: 0.1 mm. Abbreviations: DTA – dorsal tibial apophysis, E – embolus, FA – femoral apophysis, RTA – retrolateral tibial apophysis, rTA – retrolateral tegular apophysis.

The new species differs from *O. fabiformis* Liu, Xu, Xiao, Yin & Peng, 2019 and *O. hippocampa* Jin, Fu, Yin & Zhang, 2016 by the short hook-shaped embolus (Figs 9D, 11A, B) (vs. spine-like in *O. fabiformis* and *O. hippocampa*), and the C-shaped spermathecae (Fig. 10D) (vs. peanut-like in *O. fabiformis* and globular in *O. hippocampa*).

##### Description.

**Male** (holotype). Habitus as in Fig. 9A, B. Total length 2.56, carapace 1.24 long, 1.07 wide. ***Eye*** sizes and interdistances: AME 0.06, ALE 0.08, PME 0.08, PLE 0.08; ALE−AME 0.01, AME–AME 0.04, PLE−PME 0.04, PME–PME 0.07, ALE−ALE 0.17, PLE−PLE 0.30, ALE−PLE 0.07, AME−PME 0.07, ALE−PME 0.07. MOA 0.21 long, front width 0.15, back width 0.22. ***Chelicerae*** (Fig. 9B) three promarginal (proximal largest, distal smallest) and two retromarginal teeth (distal larger Sternum, posteriorly pointed. ***Abdomen*** (Fig. 9A, B), 1.42 long, 0.97 wide. Leg measurements: I 4.64 (1.21, 0.50, 1.35, 1.08, 0.50); II 4.00 (1.10, 0.48, 1.05, 0.95, 0.42); III 3.49 (0.90, 0.41, 0.74, 0.93, 0.51); IV 4.95 (1.30, 0.46, 1.10, 1.38, 0.71). ***Leg*** spination: femur I with two dorsal spines, femora II−IV with one dorsal spine each; femora I pv111, II pv11; tibiae I v2222222, II v222222; metatarsi I v2222, II v1222.

***Colouration*** (Fig. 9A, B). Carapace yellow, with radial, irregular dark stripes submarginally and arc-shaped dark stripes around margin. Chelicerae yellow brown. Endites, labium and sternum yellow. Legs yellow, with distinct annulations on tibiae and distal part of femora, patellae and metatarsi. Abdomen yellowish, with two large C-shaped stripes on the two sides of dorsal scutum and four light chevron-shaped stripes in submedial part, and single yellowish transverse stripe posteriorly.

***Palp*** (Figs 9C−F, 11). Femoral apophysis well-developed, width longer than half of length. Patella unmodified. Retrolateral tibial apophysis large, longer than tibia, horn-shaped, with a sharp apex in retrolateral view. Dorsal tibial apophysis large, slightly shorter than tibia, with sharp narrowed sub-medial part and a spine-like apex in dorsal view. Sperm duct strongly sclerotised, hook-shaped in ventral view, anterior part thick, gradually narrowed in posterior part. Retrolateral tegular apophysis extruding laterally, in front of anterior part of sperm duct. Embolus short and hook-shaped.

**Female.** Habitus as in Fig. 10A, B. Lighter than males. Total length 2.62, carapace length 1.26, width 1.10. ***Eye*** diameters: AME 0.06, ALE 0.08, PME 0.07, PLE 0.08; interdistances: ALE−AME 0.01, AME–AME 0.03, PLE−PME 0.05, PME–PME 0.07, ALE−ALE 0.14, PLE−PLE 0.31, ALE−PLE 0.08, AME−PME 0.08, ALE−PME 0.09. MOA 0.20 long, front width 0.12, back width 0.21. Sternum, posterior end proper blunt. ***Abdomen*** (Fig. 10A, B) length 1.42, width 0.89. Leg measurements: I broken; II 3.93 (1.05, 0.45, 1.06, 0.94, 0.43); III broken; IV 4.92 (1.31, 0.44, 1.17, 1.35, 0.65). ***Leg*** spination: femur I with two dorsal spines, femora II−IV with one dorsal spine each; femur II pv11.

***Colouration*** (Fig. 10A, B). Legs without distinct annulations on femora, patellae, tibiae and metatarsi. Abdomen, antero-medially with longitudinal grey-brown stripe connecting with paired yellowish spots in dorsal view.

***Epigyne*** (Fig. 10C, D). Epigynal plate snake-like, with a narrowed median septum, copulatory ducts, connecting tubes and spermathecae distinctly visible through integument in intact epigyne. Anteromedially with small round copulatory openings. Copulatory ducts short, proper broad, almost parallel, medially located between copulatory openings and glandular appendage. Connecting tubes short, C-shaped, shorter than connecting tubes. Spermathecae, C-shaped. Fertilisation ducts extending anteriorly.

##### Distribution.

Known only from the type locality in Jiangxi Province, China (Map 2).

#### Otacilia longtanica

Taxon classificationAnimaliaAraneaePhrurolithidae

Liu sp. nov.

EE616617-D7AE-518A-BFAB-4AC2C0248916

http://zoobank.org/3E6CC983-C836-4156-8D66-571ABBC64FAD

Figure 12

##### Type material.

***Holotype***: ♀, China: Jiangxi Province, Ji’an City, Jinggangshan County Level City, Ciping Town, Xiaojing Village, Longtan Scenic Spot, 26°35'56.4"N, 114°8'24.0"E, 838 m, 31 May 2014, leg. Ke-Ke Liu et al.

##### Etymology.

The specific name refers to the type locality, Longtan; adjective.

##### Differential diagnosis.

**Figure 12.**
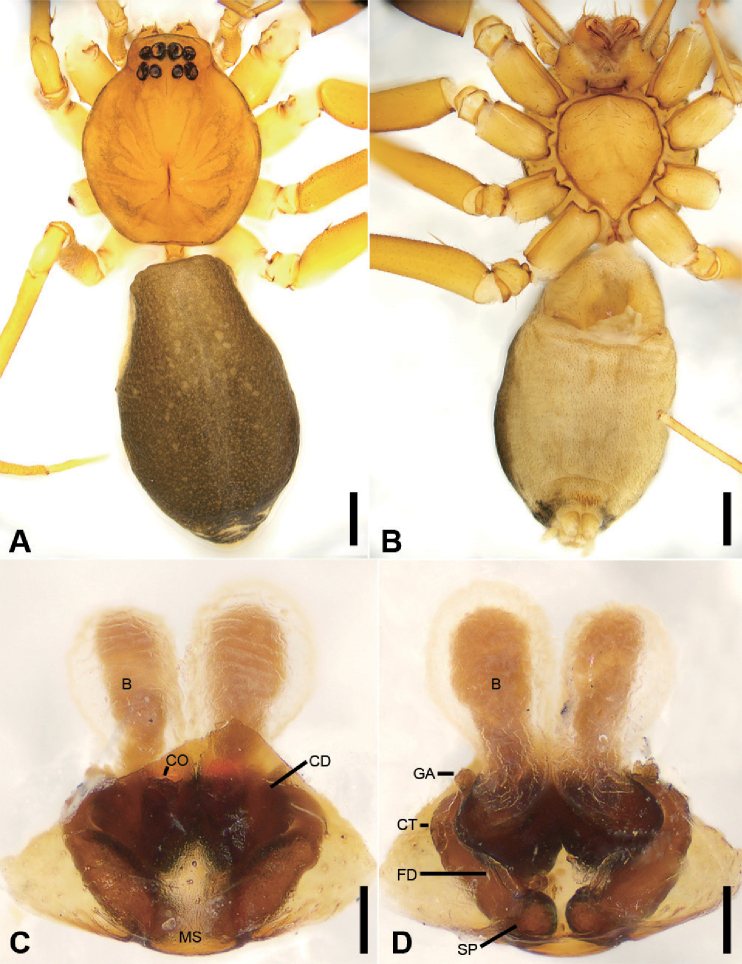
*Otacilia longtanica* sp. nov., female holotype **A** habitus, dorsal view **B** same, ventral view **C** epigyne, ventral view **D** same, dorsal view. Scale bars: 0.5 mm (**A, B**), 0.1 mm (**C, D**). Abbreviations: B – bursa, CD – copulatory duct, CO – copulatory opening, CT – connecting tube, FD – fertilisation ducts, GA – glandular appendage, MS – median septum, SP – spermathecae.

The female of this species is similar to that of *O. fujiana* Fu, Jin & Zhang, 2014 but differs by the chelicerae having two retromarginal teeth (Fig. 12B) (vs. five retromarginal teeth) and the oval spermathecae (vs. with clavate shafts). Male unknown.

##### Description.

**Female.** Habitus as in Fig. 12A, B. Total length 4.81, carapace 1.69 long, 1.01 wide. ***Eye*** sizes and interdistances: AME 0.11, ALE 0.11, PME 0.09, PLE 0.10; ALE−AME 0.04, AME–AME 0.08, PLE−PME 0.08, PME–PME 0.13, ALE−ALE 0.34, PLE−PLE 0.45, ALE−PLE 0.13, AME−PME 0.11, ALE−PME 0.18. MOA 0.32 long, front width 0.30, back width 0.32. ***Chelicerae*** (Fig. 12A) with three promarginal (middle largest, distal smallest) and two retromarginal teeth (distal larger). ***Sternum*** (Fig. 12B), with distinct precoxal triangles, posterior pointed. ***Abdomen*** (Fig. 12A) 2.70 long, 1.66 wide. ***Leg*** measurements: I 9.66 (2.41, 0.71, 3.17, 1.89, 1.48); II 7.91 (1.99, 0.62, 2.32, 1.68, 1.30); III 6.20 (1.50, 0.58, 1.56, 1.57, 0.99); IV 9.81 (2.45, 0.69, 2.51, 2.71, 1.45). Leg spination: femora I–IV with one dorsal spine each; femora I pv111111, II pv111; tibiae I v2222222222, II v22222222; metatarsi I v2222, II v1222.

***Colouration*** (Fig. 12A, B). Carapace yellow to yellow-brown, with radial, irregular dark stripes mediolaterally and arch-shaped dark stripes around margin. Chelicerae yellow. Endites yellow. Labium yellow-brown. Sternum yellow, with yellow-brown margin. Legs yellow, without annulations on tibiae and distal part of femora, patellae and metatarsi (Fig. 12A, B). Abdomen dark brown, with abundant yellowish spots in dorsal view.

***Epigyne*** (Fig. 12C, D). Epigynal plate trapezoid, antero-medially with pair of slit-like copulatory openings, with a narrowed median septum, copulatory ducts, glandular appendage, connecting tubes and spermathecae distinctly visible through integument in intact epigyne. Copulatory ducts very short, relative broad, between copulatory openings and glandular appendage, with pair of elongated transparent bursae anteriorly. Glandular appendages short, proper thick, located on the anterior of connecting tubes. Connecting tubes short, as long as copulatory duct, broad, located between glandular appendages and spermathecae. Spermathecae elongated, oval, slightly separated at their apex. Fertilisation duct short, located apically on spermathecae.

##### Distribution.

Known only from the type locality in Jiangxi Province, China (Map 2).

#### Otacilia ovoidea

Taxon classificationAnimaliaAraneaePhrurolithidae

Liu sp. nov.

43073804-DA0E-5F53-9E7B-5DC46A8F9FA1

http://zoobank.org/1F47C8A6-95FB-4B9A-9994-4E78949142A6

Figures 13 , 14

##### Type material.

***Holotype***: ♂, China: Jiangxi Province, Ji’an City, Jinggangshan County Level City, Ciping Town, Dajing Village, Jingzhushan Scenic Spot, 26°32'39.69"N, 114°06'34.96"E, 1130 m, 1 October 2018, leg. Ke-Ke Liu et al. ***Paratypes***: 7♂, 1♀, with same data as holotype; 1♂, 26°31'33.37"N, 114°06'30.34"E, 786 m, other data as holotype; 2♂, 26°32'39.69"N, 114°06'34.96"E, 1130 m, other data as holotype; 9♂, Ciping Town, Wuzhifeng Scenic Spot, 26°31'59.07"N, 114°08'28.47"E, 735 m, 2 October 2018, leg. Ke-Ke Liu et al.; 9♂, Ciping Town, Liping Village, around the Shiyan Cave, 26°36'10.43"N, 114°12'46.35"E, 955 m, 6 October 2018, leg. Ke-Ke Liu and Hui-Pu Luo; 1♂, Luofu Town, Xiangzhou Village, Fengshuping Group, 26°36'10.31"N, 114°06'34.69"E, 364 m, 5 October 2018, leg. Ke-Ke Liu and Hui-Pu Luo.

##### Etymology.

The specific name is derived from the Latin word *ovoideus*, referring to the ovoid terminal apophysis of the male palp; adjective.

##### Diagnosis.

**Figure 13.**
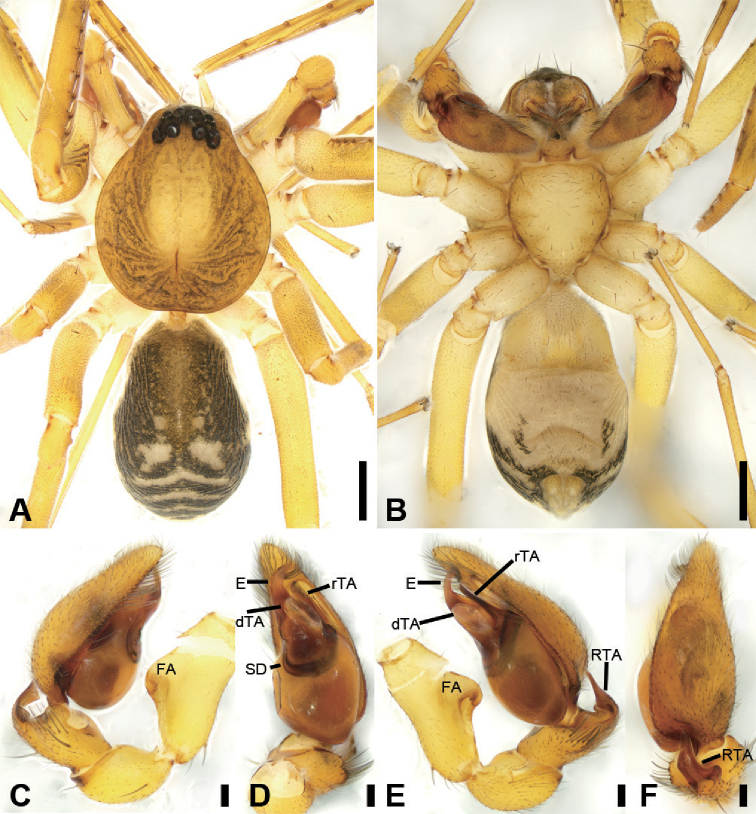
*Otacilia ovoidea* sp. nov., male holotype **A** habitus, dorsal view **B** same, ventral view **C** palp, prolateral view **D** same, ventral view **E** same, retrolateral view **F** same, dorsal view. Scale bars: 0.5 mm (**A, B**), 0.1 mm (**C–F**). Abbreviations: dTA – distal tegular apophysis, E – embolus, FA – femoral apophysis, rTA – retrolateral tegular apophysis, RTA – retrolateral tibial apophysis, SD – sperm duct.

**Figure 14.**
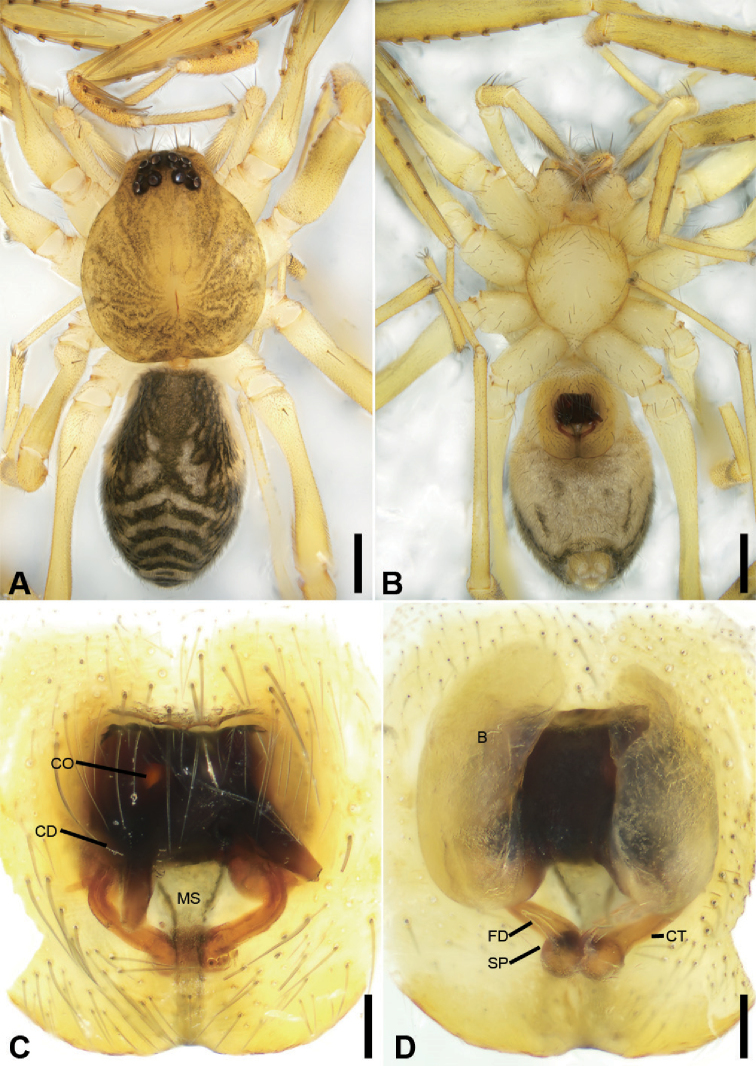
*Otacilia ovoidea* sp. nov., female paratype **A** habitus, dorsal view **B** same, ventral view **C** epigyne, ventral view **D** epigyne, dorsal view. Scale bars: 0.5 mm (**A, B**), 0.1 mm (**C, D**). Abbreviations: B – bursa, CD – copulatory duct, CO – copulatory opening, CT – connecting tube, FD – fertilisation ducts, GA – glandular appendage, MS – median septum, SP – spermathecae.

This species can be easily recognised by the palp (Fig. 13C−F) with the clavate retrolateral tegular apophysis (vs. absent, triangular, finger-shaped, or otherwise) and the ovoid membranous fan-shaped distal tegular apophysis (Fig. 13D, E) (vs. absent, ovoid, triangular, finger-shaped, or otherwise). Females are distinguished by the epigyne (Fig. 14C, D) with a weakly sclerotised transversal margin (vs. absent, M-shaped, arc-shaped, or otherwise), the funnel-shaped median septum (vs. rectangular, triangular, others), and the touching globular spermathecae (vs. widely or slightly separated).

##### Description.

**Male** (holotype). Habitus as in Fig. 13A, B. Total length 3.55, carapace 1.65 long, 1.42 wide. ***Eye*** sizes and interdistances: AME 0.10, ALE 0.10, PME 0.09, PLE 0.10; ALE−AME 0.01, AME–AME 0.05, PLE−PME 0.07, PME–PME 0.11, ALE−ALE 0.25, PLE−PLE 0.41, ALE−PLE 0.10, AME−PME 0.08, ALE−PME 0.17. MOA 0.25 long, front width 0.23, back width 0.29. ***Chelicerae*** (Fig. 13A, B) with three promarginal (middle largest, distal smallest) and six retromarginal teeth (distal largest, proximal smallest). Sternum (Fig. 13B) gradually pointed. ***Abdomen*** (Fig. 13A, B) 1.69 long, 1.01 wide. ***Leg*** measurements: I 7.10 (1.82, 0.65, 2.10, 1.71, 0.82); II 5.85 (1.53, 0.61, 1.61, 1.34, 0.76,); III 4.82 (1.27, 0.49, 1.07, 1.28, 0.71); IV 7.47 (1.99, 0.66, 1.82, 2.16, 0.84). Leg spination: femur I with two dorsal spines, femora II−IV with one dorsal spine each; femora I pv1111 (right), pv11111, II pv111; tibiae I v22222222, II v2222222; metatarsi I v2222, II v2222.

***Colouration*** (Fig. 13A, B). Prosoma yellow-brown, with radial, irregular dark brown mottled markings in the surface. Fovea distinct, black. Chelicerae yellow-brown. Endites, labium and sternum yellow. Legs yellow (Fig. 13A, B). Abdomen dark brown, with pair of round and Y-shaped spots located in the posterior dorsal scutum and three light chevron-shaped stripes on posterior part, with yellowish transversal stripe in front of the anal tubercle.

***Palp*** (Fig. 13C−F). Femoral apophysis well-developed, width longer than half of length. Patella unmodified. Retrolateral tibial apophysis large, bending inward to the base of cymbium, triangular extruding in proximal part in retrolateral view, with a clear apophyses located at the base and a blunt apex in dorsal view. Sperm duct C-shaped, strongly sclerotised, around the base of retrolateral tegular apophysis, distal tegular apophysis and embolus; distal tegular apophysis club-shaped, longer than embolus. Conductor, ovoid, slightly shorter than embolus. Embolus, with proper broad base and a short, curved tip.

**Female.** Habitus as in Fig. 14A, B. Total length 3.73, carapace 1.77 long, 1.57 wide. ***Eye*** sizes and interdistances: AME 0.08, ALE 0.08, PME 0.08, PLE 0.10; ALE−AME 0.03, AME–AME 0.07, PLE−PME 0.08, PME–PME 0.15, ALE−ALE 0.28, PLE−PLE 0.46, ALE−PLE 0.12, AME−PME 0.10, ALE−PME 0.11. MOA 0.26 long, front width 0.23, back width 0.31. ***Abdomen*** (Fig. 14A, B) 1.90 long, 1.20 wide. ***Leg*** measurements: I 7.36 (1.80, 0.65, 2.30, 1.75, 0.86); II 5.85 (1.45, 0.62, 1.67, 1.29, 0.82); III 5.12 (1.38, 0.56, 1.12, 1.31, 0.75); IV 7.73 (2.12, 0.66, 1.79, 2.06, 1.10). Leg spination: femur I with two dorsal spines, femora II−IV with one dorsal spine each; femur I pv1111; tibiae I v22222222, II v2222222; metatarsus II v1222.

***Epigyne*** (Fig. 14C, D). Epigynal plate bow-shaped, antero-medially with pair of concaved copulatory openings, with a funnel-shaped median septum, copulatory ducts, glandular appendage, connecting tubes and spermathecae distinctly visible through integument in intact epigyne. Anterior fovea separated by weakly sclerotised transversal margin. Copulatory ducts broad, located between copulatory openings and glandular appendages, posteriorly with pair of large, oval, transparent bursae. Glandular appendages short, partly covered by bursae, located on the anterior of connecting tubes. Connecting tubes slightly shorter than copulatory ducts, located between glandular appendages and spermathecae. Spermathecae globular, directed medially. Fertilisation duct short, located apically on spermathecae.

##### Distribution.

Known only from the type locality in Jiangxi Province, China (Map 2).

#### Otacilia shenshanica

Taxon classificationAnimaliaAraneaePhrurolithidae

Liu sp. nov.

3B2C98BE-CFCF-5366-AB5C-4B7C09D3117B

http://zoobank.org/354A0C02-F10E-4B37-94AB-2FFDCA6F3EB2

**Figure 15.**
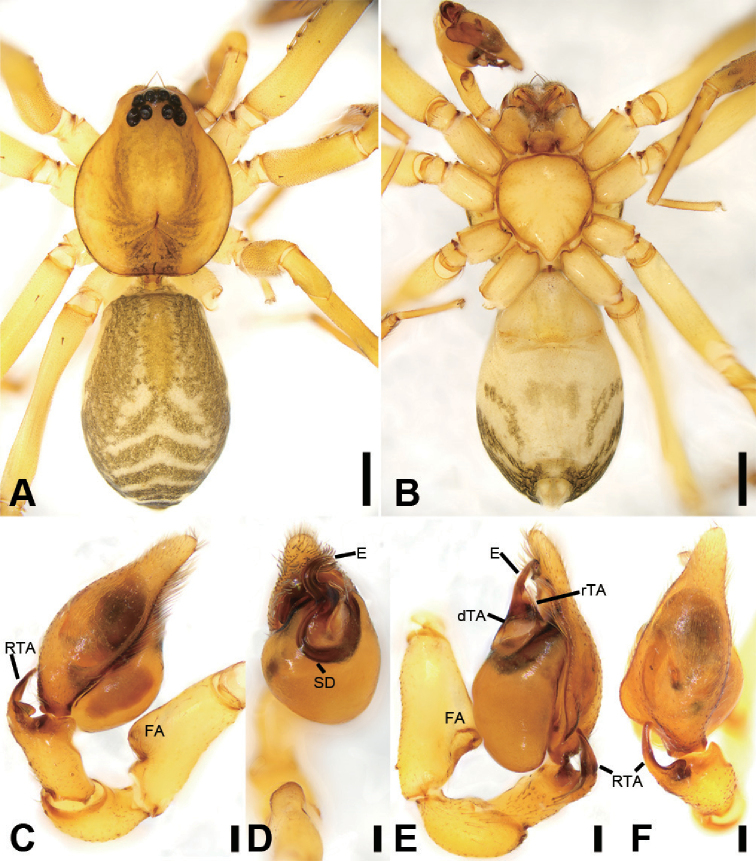
*Otacilia shenshanica* sp. nov., male holotype **A** habitus, dorsal view **B** same, ventral view **C** palp, prolateral view **D** same, ventral-distal view **E** same, retrolateral view **F** same, dorsal view. Scale bars: 0.5 mm (**A, B**), 0.1 mm (**C–F**). Abbreviations: dTA – distal tegular apophysis, E – embolus, FA – femoral apophysis, RTA – retrolateral tibial apophysis, rTA – retrolateral tegular apophysis, SD – sperm duct.

**Figure 16.**
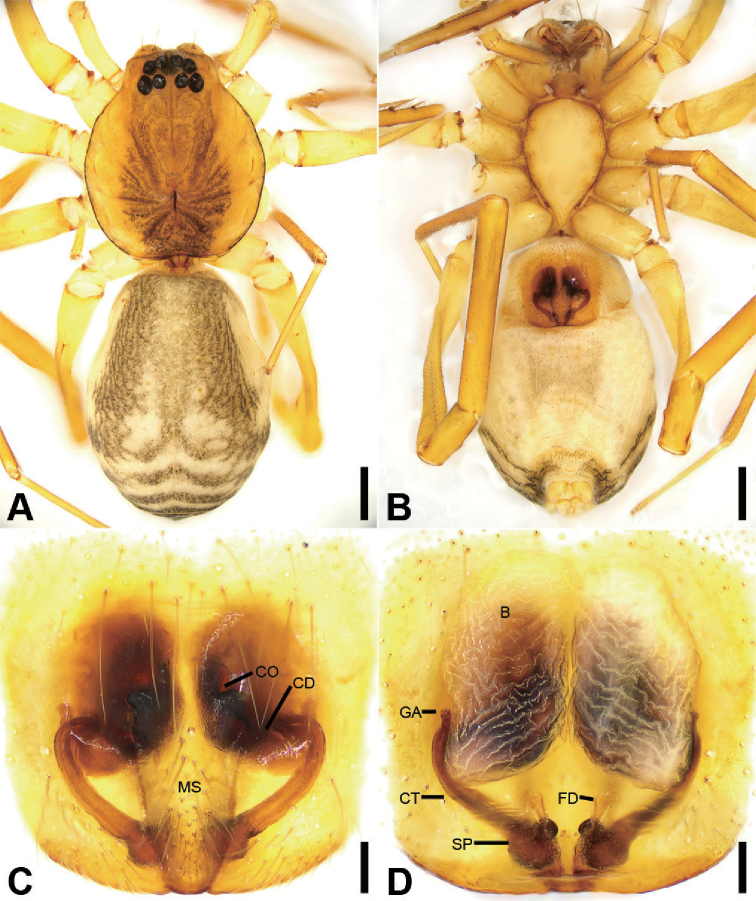
*Otacilia shenshanica* sp. nov., female paratype **A** habitus, dorsal view **B** same, ventral view **C** epigyne, ventral view **D** epigyne, dorsal view. Scale bars: 0.5 mm (**A, B**), 0.1 mm (**C, D**). Abbreviations: B – bursa, CD – copulatory duct, CO – copulatory opening, CT – connecting tube, FD – fertilisation ducts, GA – glandular appendage, MS – median septum, SP – spermathecae.

**Figure 17.**
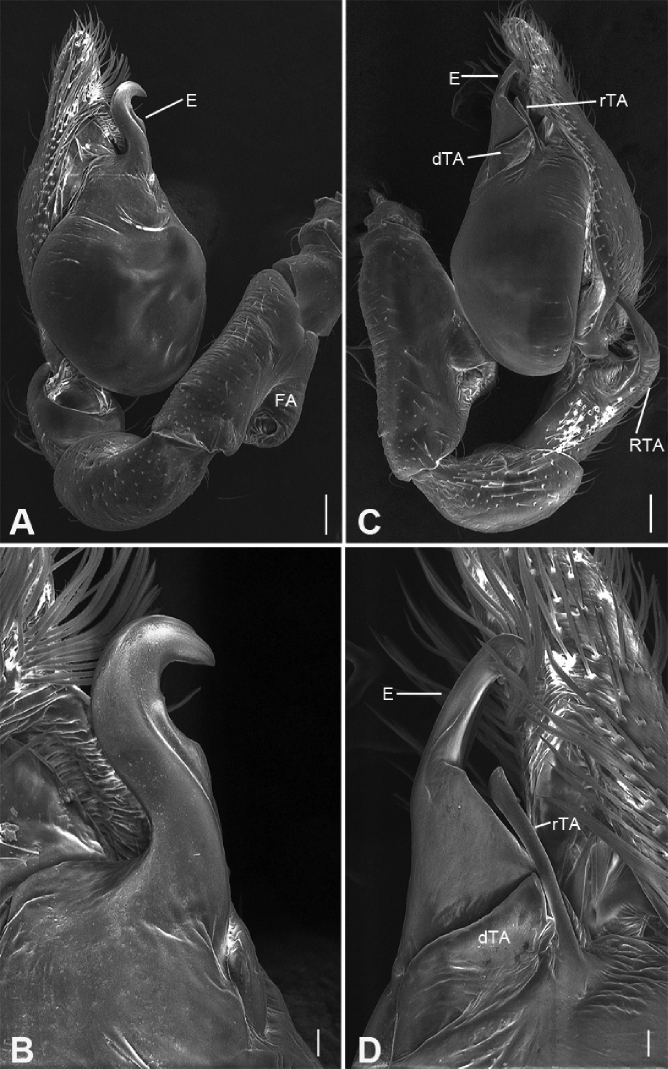
SEM micrographs of *Otacilia shenshanica* sp. nov., palp of male paratype **A** proventral view **B** same, detail showing embolus **C** retrolateral view **D** same, detail of conductor, embolus and tegular apophysis. Scale bars: 0.1 mm (**A, C**), 20 µm (**B, D**). Abbreviations: dTA – distal tegular apophysis, E – embolus, FA – femoral apophysis , RTA – retrolateral tibial apophysis, rTA – retrolateral tegular apophysis.

Figures 15 , 16 , 17

##### Type material.

***Holotype***: ♂, China: Jiangxi Province, Ji’an City, Jinggangshan County Level City, Dalong Town, Yuantou Village, 26°37'55.2"N, 114°06'21.6"E, 1029 m, 5 April 2014, leg. Ke-Ke Liu et al. ***Paratypes***: ♀, with same data as holotype; 1♂, 2♀, 26°37'33.6"N, 114°06'21.6"E, 791 m, other data as holotype; 1♀, Longshi Town, Maoping, Shenshan Village, Shenshan, 26°38'13.2"N, 114°06'39.6"E, 1099 m, 6 April 2014, leg. Ke-Ke Liu et al.

##### Etymology.

The specific name refers to the type locality, Shenshan; adjective.

##### Differential diagnosis.

The new species differs from *O. hengshan* (Song, 1990) by the bend of the RTA with a strong basal apophysis (Figs 15C, E, F, 17C) (vs. the sub-median part of the RTA with a strong apophysis) and the wider median septum located medially (Fig. 16C, D) (vs. narrowed).

##### Description.

**Male** (holotype). Habitus as in Fig. 15A. Total length 3.87, carapace 1.72 long, 1.45 wide. ***Eye*** sizes and interdistances: AME 0.08, ALE 0.10, PME 0.08, PLE 0.09; ALE−AME 0.03, AME–AME 0.06, PLE−PME 0.07, PME–PME 0.14, ALE−ALE 0.27, PLE−PLE 0.41, ALE−PLE 0.11, AME−PME 0.10, ALE−PME 0.19. MOA 0.26 long, front width 0.21, back width 0.29. Cervical groove and fovea distinct. ***Chelicerae*** (Fig. 15A, B) with three promarginal (middle largest, distal smallest) and six retromarginal teeth (distal largest, proximal smallest). ***Sternum*** (Fig. 15B), posterior pointed. ***Abdomen*** (Fig. 15A, B) 1.98 long, 1.38 wide, weak dorsal scutum in anterior half. ***Leg*** measurements: I 7.21 (1.87, 0.70, 2.24, 1.77, 0.63); II 5.52 (1.52, 0.58, 1.45, 1.38, 0.59); III 4.86 (1.31, 0.54, 1.08, 1.33, 0.60); IV 7.77 (2.09, 0.63, 1.88, 2.15, 1.02). Leg spination: femur I with two dorsal spines, femora II−IV with one dorsal spine each; femora I pv1111 (right), pv11111, II pv111; tibiae I v22222222, II v222222; metatarsi I v2222, II v1222.

***Colouration*** (Fig. 15A, B). Carapace yellow, with radial, irregular dark stripes medially and arch-shaped dark stripes around margin. Chelicerae yellow-brown. Endites yellow. Labium yellow-brown. Sternum yellow. Legs yellow, without annulations on tibiae and distal part of femora, patellae, and metatarsi. Abdomen dark brown, with pair of oval and pair of clavate yellowish spots on the posterior dorsal scutum, three light chevron-shaped stripes in posterior part, and yellowish arch-shaped stripe in front of the anal tubercle.

***Palp*** (Figs 15C−F, 17). Femoral apophysis well developed, width less than half of length. Patella unmodified. Retrolateral tibial apophysis large, slightly less than tibia, finger-like, bending inwards towards base of cymbium, with strong basal apophysis and blunt tip. Sperm duct O-shaped, strongly sclerotised, around base of retrolateral tegular apophysis, distal tegular apophysis and embolus. Retrolateral tegular apophysis clavate, slightly shorter than embolus. Distal tegular apophysis triangular, accompanied by embolus and subterminal apophysis. Embolus, thick, hook-shaped, with broad base and blunt tip.

**Female.** Habitus as in Fig. 16A, B. Darker than males. Total length 4.35, carapace 1.91 long, 1.67 wide. ***Eye*** sizes and interdistances: AME 0.10, ALE 0.11, PME 0.09, PLE 0.10; ALE−AME 0.02 AME–AME 0.07, PLE−PME 0.08, PME–PME 0.14, ALE−ALE 0.30, PLE−PLE 0.47, ALE−PLE 0.11, AME−PME 0.11, ALE−PME 0.11. MOA 0.28 long, front width 0.25, back width 0.33. ***Abdomen*** (Fig. 16A) 2.27 long, 1.73 wide. ***Legs*** (Fig. 13A) measurements: I 7.84 (2.03, 0.75, 2.39, 1.83, 0.84); II 6.61 (1.74, 0.66, 1.87, 1.51, 0.83); III 5.52 (1.43, 0.62, 1.34, 1.42, 0.71); IV 8.39 (2.23, 0.74, 2.01, 2.33, 1.08). Leg spination: femur I pv1111; tibia II v22222222.

***Colouration*** (Fig. 16A, B). Abdomen with pair of irregular yellowish spots behind the first pair of oval spots.

***Epigyne*** (Fig. 16C, D). Epigynal plate bow-shaped, antero-medially with pair of concaved copulatory openings, with triangular median septum, copulatory ducts, glandular appendage, connecting tubes and spermathecae distinctly visible through integument in intact epigyne. Copulatory ducts broad, slightly sloping, located between copulatory openings and glandular appendages, posteriorly with pair of large, bean-shaped, transparent bursae. Glandular appendages short, partly covered by bursae, located on anterior of connecting tubes. Connecting tubes, twice the length of copulatory ducts, located between glandular appendages and spermathecae. Spermathecae globular, slightly separated. Fertilisation duct short, located apically on spermathecae, extending anteriorly.

##### Distribution.

Known only from the type locality in Jiangxi Province, China (Map 2).

#### Otacilia subovoidea

Taxon classificationAnimaliaAraneaePhrurolithidae

Liu sp. nov.

9C3DA71D-B0C6-50A4-9969-5955233DC432

http://zoobank.org/B862C4C7-C715-4DE1-B3A3-7A1C01B54518

**Figure 18.**
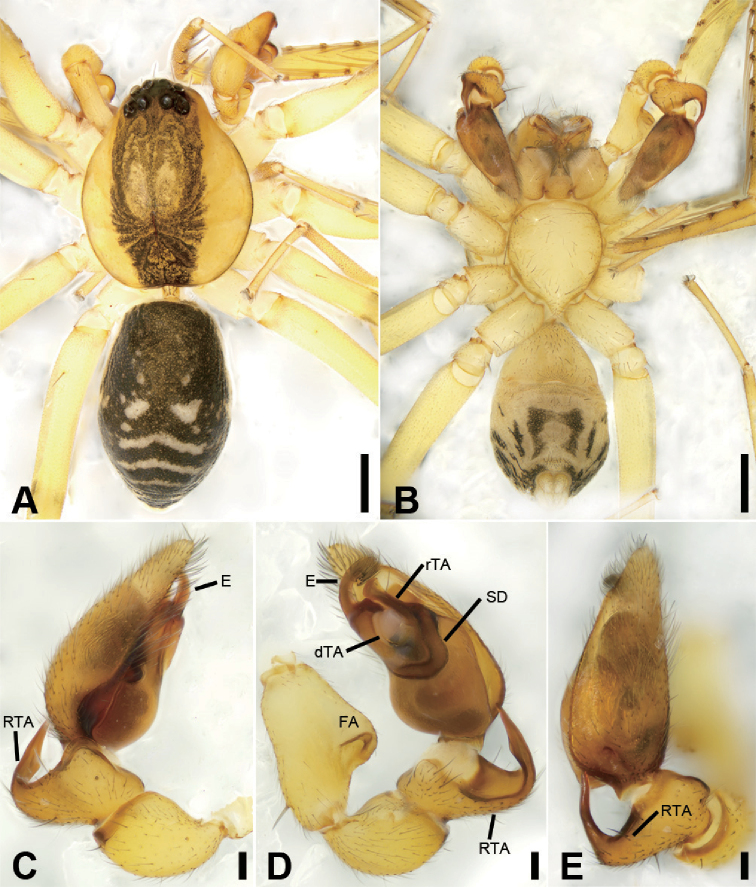
*Otacilia subovoidea* sp. nov., male holotype **A** habitus, dorsal view **B** same, ventral view **C** palp, prolateral view **D** same, ventral view **E** same, dorsal view. Scale bars: 0.5 mm (**A, B**), 0.1 mm (**C–E**). Abbreviations: dTA – distal tegular apophysis, E – embolus, FA – femoral apophysis, RTA – retrolateral tibial apophysis, rTA – retrolateral tegular apophysis, SD – sperm duct.

**Figure 19.**
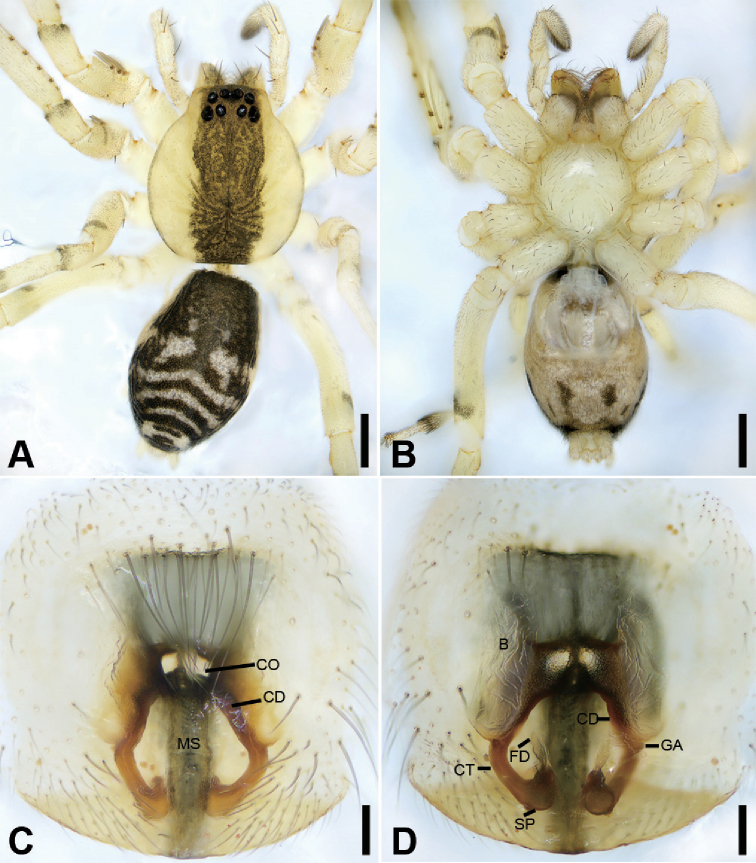
*Otacilia subovoidea* sp. nov., female paratype **A** habitus, dorsal view **B** same, ventral view **C** epigyne, ventral view **D** epigyne, dorsal view. Scale bars: 0.5 mm (**A, B**), 0.1 mm (**C, D**). Abbreviations: B – bursa, CD – copulatory duct, CO – copulatory opening, CT – connecting tube, FD – fertilisation ducts, GA – glandular appendage, MS – median septum, SP – spermathecae.

**Figure 20.**
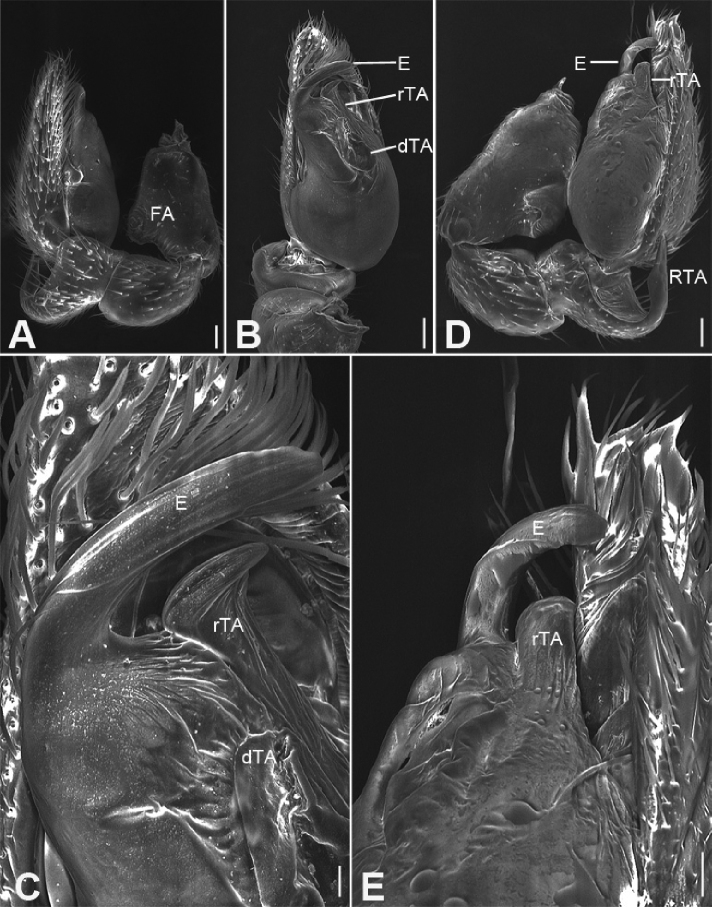
SEM micrographs of *Otacilia subovoidea* sp. nov., male paratype **A** palp, prolateral view **B** same, ventral view **C** same, ventral view, detail of conductor, embolus and tegular apophysis **D** same, retrolateral view **F** same, retrolateral view, detail of embolus and tegular apophysis. Scale bars: 0.1 mm (**A, B, D**), 20 µm (**C**), 40 µm (**E**). Abbreviations: dTA – distal tegular apophysis, E – embolus, FA – femoral apophysis, RTA – retrolateral tibial apophysis, rTA – retrolateral tegular apophysis.

Figures 18 , 19 , 20

##### Type material.

***Holotype***: ♂, China: Jiangxi Province, Ji’an City, Jinggangshan County Level City, Ciping Town, Liping Village, Citic Sewage Treatment Plant, 26°35'28.93"N, 114°12'46.82"E, 810 m, 6 October 2018, leg. Ke-Ke Liu and Hui-Pu Luo. ***Paratypes***: 6♂, 3♀, with same data as holotype; 4♂, 5♀, Liping Village, around the Shiyan Cave, 26°36'13.60"N, 114°12'35.91"E, 927 m, other data as holotype; 2♂, 2♀, Dajing Village, Lingxiufeng Scenic Spot, 26°34'16.72"N, 114°07'00.56"E, 971 m, 1 October 2018, leg. Ke-Ke Liu et al.; 5♂, Xiaojing Village, Longtan Scenic Spot, 26°35'33.08"N, 114°08'18.50"E, 909 m, 1 October 2018, leg. Ke-Ke Liu et al.; 2♂, 3♀, Wuzhifeng Scenic Spot, 26°31'59.07"N, 114°08'28.47"E, 735 m, 2 October 2018, leg. Ke-Ke Liu et al.; 3♀, Jingzhushan Scenic Spot, 26°32'39.69"N, 114°06'34.96"E, 1130 m, 1 October 2018, leg. Ke-Ke Liu et al.; 2♂, 3♀, Wuzhifeng Scenic Spot, 26°32'48.23"N, 114°09'10.61"E, 811 m, 2 October 2018, leg. Ke-Ke Liu et al.

##### Etymology.

The specific name is derived from that of a similar species, *O. ovoidea* sp. nov.; adjective.

##### Diagnosis.

The new species differs from *O. ovoidea* sp. nov. by the relatively longer spine-like tip of embolus (Figs 18D, 20) (vs. short, hook-shaped), the straight broad retrolateral tegular apophysis (Figs 18D, 20) (vs. thin, clavate) and by the relatively broader bar-shaped median septum (Fig. 19C) (vs. funnel-shaped, anteriorly broad, posteriorly thin), and the separated spermathecae (Fig. 19D) (vs. touching spermathecae).

##### Description.

**Male** (holotype). Habitus as in Fig. 18A, B. Total length 3.44, carapace 1.69 long, width 1.44 wide. ***Eye*** sizes and interdistances: AME 0.10, ALE 0.10, PME 0.07, PLE 0.11; ALE−AME 0.02, AME–AME 0.06, PLE−PME 0.09, PME–PME 0.14, ALE−ALE 0.26, PLE−PLE 0.44, ALE−PLE 0.11, AME−PME 0.11, ALE−PME 0.19. MOA 0.25 long, front width 0.22, back width 0.29. ***Chelicerae*** (Fig. 18A, B) with three promarginal (proximal largest, distal smallest) and six retromarginal teeth (distal largest, proximal smallest). ***Sternum*** (Fig. 18B) longer than wide. ***Abdomen*** (Fig. 18A, B) 1.66 long, 1.01 wide. ***Leg*** measurements: I 7.00 (1.79, 0.63, 2.13, 1.69, 0.76); II 5.76 (1.50, 0.58, 1.60, 1.33, 0.75); III 4.30 (1.25, 0.53, 0.90, 1.03, 0.59); IV 7.48 (2.02, 0.60, 1.84, 2.10, 0.91). Leg spination: femur I with two dorsal spines, femora II−IV with one dorsal spine each; femora I pv1111, II pv11; tibiae I v22222222, II v222222; metatarsi I v2222, II pv1222.

***Colouration*** (Figs 18A, B). Carapace yellow, medially with broad dark brown mottled markings in the surface. Fovea distinct, black. Chelicerae, endites, labium and sternum yellow brown. Legs yellow, without dark annulation. Abdomen dark brown, with pair of round and oval pale spots located in the posterior dorsal scutum and three light chevron-shaped stripes in posterior part, and one yellowish transversal stripe in front of the anal tubercle.

***Palp*** (Figs 18C−E, 20). Femoral apophysis well-developed, width more than half of length. Patella unmodified. Retrolateral tibial apophysis large, longer than tibia, sword-like in ventral view, bending inward to the base of cymbium, medial part widened and slightly curved, with a strong spine-like tip. Sperm duct U-shaped, strongly sclerotised, around the base of subterminal apophysis, terminal apophysis and embolus. Subterminal apophysis, straight, broad, as long as embolus, anteriorly widened. Terminal apophysis, membranous, fan-shaped, extending to median bulb. Embolus, thick, hook-shaped, with a broad base and a blunt tip. Embolus relatively long, thick spine like, with broad base and a blunt apex.

**Female.** Habitus as in Fig. 19A, B. Lighter than males. Total length 3.57, carapace 1.66 long, 1.46 wide. ***Eye*** sizes and interdistances: AME 0.07, ALE 0.07, PME 0.07, PLE 0.09; ALE−AME 0.04, AME–AME 0.08, PLE−PME 0.09, PME–PME 0.14, ALE−ALE 0.28, PLE−PLE 0.43, ALE−PLE 0.12, AME−PME 0.13, AME−PLE 0.11. MOA 0.26 long, front width 0.21, back width 0.27. ***Abdomen*** (Fig. 19A, B) 1.80 long, 1.15 wide. ***Leg*** measurements: I 7.12 (1.81, 0.68, 2.21, 1.67, 0.75); II 5.76 (1.50, 0.58, 1.65, 1.29, 0.74); III 4.91 (1.31, 0.48, 1.14, 1.20, 0.78); IV 7.56 (2.10, 0.66, 1.85, 2.05, 0.90). Leg spination: tibia II v22222222.

***Epigyne*** (Fig. 19C, D). Epigynal plate mask-like, anterior margin slightly sclerotised, transverse, medially with pair of touching hole-shaped copulatory openings, posteriorly with bar-shaped median septum, copulatory ducts, connecting tubes and spermathecae distinctly visible through integument in intact epigyne. Copulatory ducts between copulatory openings and glandular appendages, sloping laterally, proper broad, posteriorly with pair of large, oval, transparent bursae. Glandular appendages short, partly covered by bursae, located on anterior of connecting tubes. Connecting tubes slightly shorter than copulatory ducts, slightly curved backwards. Spermathecae sub-spherical, directed medially, separated by mark of median septum. Fertilisation duct short, with semi-ovoid base, directed forward.

##### Distribution.

Known only from the type locality in Jiangxi Province, China (Map 2).

#### Otacilia xiaoxiica

Taxon classificationAnimaliaAraneaePhrurolithidae

Liu sp. nov.

117BD41D-ECC6-5ED6-B2CC-F0D25EDBD650

http://zoobank.org/CFC851C2-2547-427C-9646-4ACD8B904229

Figure 21

##### Type material.

***Holotype***: ♂, China: Jiangxi Province, Ji’an City, Jinggangshan County Level City, Huangao Town, Xiaoxi Forest Farm, 26°28'8.4"N, 114°12'36.0"E, 365 m, 30 May 2017, leg. Ke-Ke Liu et al.

##### Etymology.

The specific name refers to the type locality, Xiaoxi Forest Farm; adjective.

##### Differential diagnosis.

**Figure 21.**
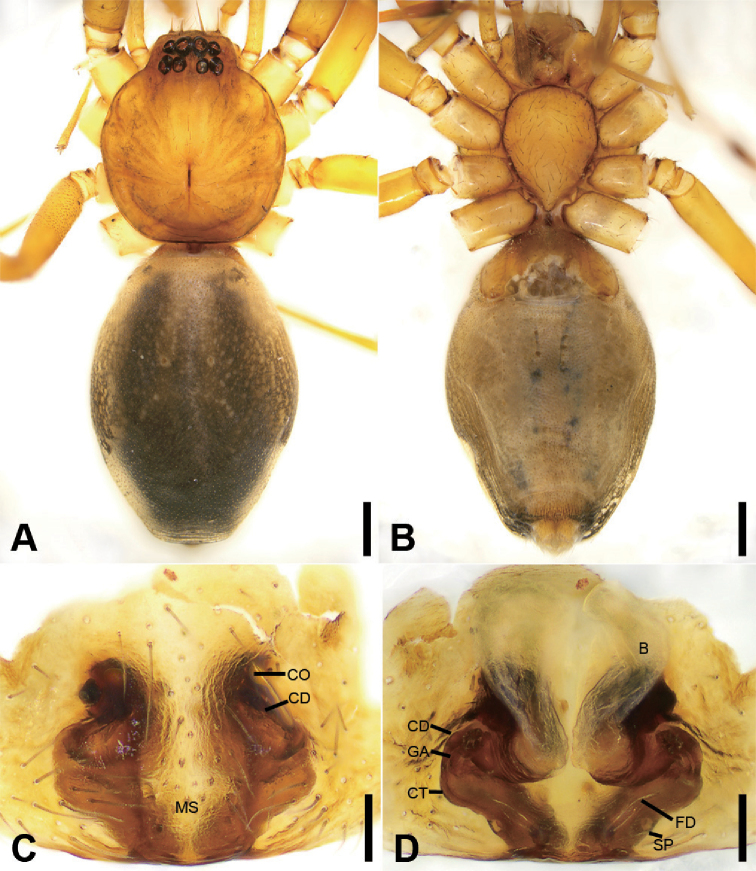
*Otacilia xiaoxiica* sp. nov., female holotype **A** habitus, dorsal view **B** same, ventral view **C** epigyne, ventral view **D** epigyne, dorsal view. Scale bars: 0.5 mm (**A, B**), 0.1 mm (**C, D**). Abbreviations: B – bursa, CD – copulatory duct, CO – copulatory opening, CT – connecting tube, FD – fertilisation ducts, GA – glandular appendage, MS – median septum, SP – spermathecae.

The female of this species differs from these of *O. fujiana* and *O. taiwanica* (Hayashi & Yoshida, 1993) by the chelicerae with three retromarginal teeth (Fig. 21B) (vs. five in *O. fujiana* and two, three or four in *O. taiwanica*) and the broad spermathecae medially with indistinct curved (Fig. 21D) (vs. the thin connecting tubes in *O. fujiana* and *O. taiwanica*, medially with distinct curve in *O. taiwanica*). Male unknown.

##### Description.

**Female.** Habitus as in Fig. 21A, B. Total length 4.79, carapace 1.97 long, 1.68 wide. ***Eye*** sizes and interdistances: AME 0.12, ALE 0.12, PME 0.10, PLE 0.12; ALE−AME 0.03, AME–AME 0.06, PLE−PME 0.07, PME–PME 0.12, ALE−ALE 0.36, PLE−PLE 0.45, ALE−PLE 0.12, AME−PME 0.10, ALE−PME 0.15. MOA 0.31 long, front width 0.30, back width 0.32. ***Chelicerae*** (Fig. 21A, B) with three promarginal (middle largest, distal smallest) and three retromarginal teeth (distal largest, proximal smallest). ***Sternum*** (Fig. 21B), posteriorly proper blunt. ***Abdomen*** (Fig. 21A, B) 2.69 long, 1.91 wide. Sternum longer than wide. Leg measurements: I 10.15 (2.41, 0.71, 3.12, 2.22, 1.63); II 7.95 (2.05, 0.63, 2.43, 1.63, 1.21); III 6.70 (1.76, 0.61, 1.58, 1.73, 1.02); IV broken. ***Leg*** spination: femora I−IV with one dorsal spine each; femora I pv111111, II pv11111; patella I rv1; tibiae I v2222222222, II v22222222; metatarsi I v2222, II pv1222.

***Colouration*** (Fig. 21A, B). Carapace yellow, with radial, irregular dark stripes mediolaterally. Sternum yellow, with yellow-brown margin. Legs yellow, without annulations on tibiae and distal part of femora, patellae and metatarsi. Abdomen brown, with abundant yellowish spots in dorsal view.

***Epigyne*** (Fig. 21C, D). Epigynal plate sub-square, anterolaterally with pair of crescent-shaped copulatory openings, medially with broad bar-shaped median septum, copulatory ducts and connecting tubes distinctly visible through integument in intact epigyne. Copulatory ducts broad, curved, posteriorly with pair of large, oval, transparent bursae. Glandular appendages relatively long, located on the anterior of connecting tubes. Connecting tube very short, posteriorly almost fused with spermathecae. Spermathecae broad, slightly separated at their apex. Fertilisation duct short, directed antero-laterally.

##### Distribution.

Known only from the type locality in Jiangxi Province, China (Map 2).

## Supplementary Material

XML Treatment for Alboculus

XML Treatment for Alboculus zhejiangensis

XML Treatment for Otacilia

XML Treatment for Otacilia acutangula

XML Treatment for Otacilia bijiashanica

XML Treatment for Otacilia longtanica

XML Treatment for Otacilia ovoidea

XML Treatment for Otacilia shenshanica

XML Treatment for Otacilia subovoidea

XML Treatment for Otacilia xiaoxiica
